# Distinct gene reprogramming in rosetted and symptomless shoots from the same mature rose plants infected with rose rosette virus

**DOI:** 10.3389/fpls.2025.1635660

**Published:** 2025-08-28

**Authors:** Reghan Mutethia, Shakil Hosain, Venura Herath, Kevin Ong, Oscar Riera-Lizarazu, David Byrne, Michael V. Kolomiets, Katherine M. Berg-Falloure, Jeff Floyd, Jeanmarie Verchot

**Affiliations:** ^1^ Department of Plant Pathology and Microbiology, Texas A&M University, College Station, TX, United States; ^2^ Department of Agricultural Biology, Faculty of Agriculture, University of, Peradeniya, Sri Lanka; ^3^ Department of Horticultural Sciences, Texas A&M University, College Station, TX, United States; ^4^ Baylor University, Waco, TX, United States

**Keywords:** rose rosette virus, emaravirus/pathogenicity, emaravirus/physiology, disease resistance, gene expression profiling, plant gene expression regulation, transcriptomics, plant virus disease

## Abstract

Rose rosette virus (RRV) causes disease in rose shrubs manifesting as abnormal branch growth, stem thickening, increased thorniness, as well as malformed, discolored leaves and flowers. The uneven and strange development near apical regions and only in parts of the plant led us to investigate how RRV influences growth promoters to alter internal developmental programs. Leaf samples were collected from symptomatic (rosetted) and asymptomatic shoots of the same rose plants. We quantified viral RNA levels and analyzed the concentrations of some key hormones (abscisic acid [ABA], caffeic acid [CFA], indole acetic acid [IAA], and gibberellin [GA]). Additionally, gene expression profiling was performed, focusing on genes involved in hormone synthesis and signaling, auxin transport, and plant development. Viral RNA levels were unevenly distributed between rosetted and non-rosetted tissues. The ABA and IAA levels were similar between tissue types, whereas CFA and GA exhibited marked differences. We identified 39 genes with distinct or opposite expression in rosetted versus asymptomatic tissues, including *PILS3, PIN1*, and two *SAUR* genes related to auxin transport and response. Expression of key regulators of ABA and GA synthesis and signaling, including *YUCCA* and *AUX/IAA* genes, were altered. Notably, Lonely Guy 3 (LOG3), which encodes a cytokinin-acitvating enzyme implicated in leaf patterning was significantly reduced in rosetted leaves, suggesting leaf-specific hormone imbalances. Several WOX transcription factors were suppressed indicating a potential role in antiviral responses. Our findings demonstrate that RRV selectively alters hormonal profiles and gene expression involved in plant growth and development. This study identified precise incursions of RRV into host molecular mechanisms controlling plant development and growth.

## Introduction

Rosette disease and witches’ broom disease are similar pathogen-induced abnormal asymmetric plant growth patterns. Rosette disease is characterized by the abnormal formation of leaves that cluster around the apical growing point of a stem. Witches’ broom disease is described as an abnormal cluster of branches that resembles a broom. According to the International Committee on Taxonomy of Viruses (ICTV) (https://ictv.global/taxonomy/) plant viruses that cause rosetting disease include the groundnut rosette virus (GRV; *Umbravirus*), the peach rosette mosaic virus (PRMV; *Nepovirus*), the turnip rosette virus (TuRV; *Sobemovirus*), and the rose rosette virus (RRV; *Emaravirus*). Blue palo verde broom virus is a species relative to RRV and causes witches broom in palo verde trees (Ilyas et al., 2018).

There remains little knowledge of how virus infection alters these complex interactions to cause such abnormalities in plant growth and reproductive development (Zhao and Li, 2021). In the case of roses which have multiple growth axes, or canes, originating from a common shank, we can simultaneously study both healthy looking (asymptomatic) and severely diseased branches of rose rosette infected plants (Devadas and Beck, 1972; Monder et al., 2023; Shamso et al., 2019; Zhang, 1992). Shoot growth, lateral and apical bud development, leaf patterning, and reproductive development are largely driven by complex networks of phytohormones, intricate signaling pathways, and gene regulatory networks that govern tissue pattern formation, organogenesis, cell fate, and responses to environmental stimuli. Taking advantage of the unique plant architecture and growth habits, this study employs genomic and transcriptomics driven investigations revealing complex interactions driven by RRV and phytohormones such as abscisic acid (ABA), indole 3-acetic acid (IAA; auxin), cytokinins (CK), gibberellins (GAs), and strigolactones (SLs) which influence specific transcription factors and epigenetic modifications controlling spatial patterning and differentiation (Schaller et al., 2015).

Rose rosette disease (RRD) in garden roses is associated with the transmission of RRV by infestations of eriophyid mites (*Phyllocoptes fructiphilus*) and is marked by abnormal bushy growth of branches, stem thickening, hyperthorniness, as well as malformed, discolored leaves and flowers (Aparicio Claros et al., 2022; Pemberton et al., 2018; Al Rwahnih et al., 2019). The typical symptoms of RRD are near the top of the shrub among newly growing lateral tissues in one or more canes, although not usually in all canes. RRV is a member of the genus *Emaravirus*, the family *Fimoviridae*, the order *Elliovirales*, and the class *Bunyaviricetes* (Kuhn et al., 2024). The negative-strand RNA genome consists of seven segments, and all, except for RNA6, which encodes two putative open reading frames (ORFs), are monocistronic. RNA1 encodes the putative RNA-dependent RNA polymerase (RdRp), RNA2 encodes the putative glycoprotein, RNA3 encodes the nucleocapsid, and RNA4 encodes a putative movement protein. The functions of proteins derived from RNA5, RNA6, and RNA7 remain unknown (Laney et al., 2011; Urrutia et al., 2022; Verchot et al., 2020). An RRV minireplicon and encapsidation system has been developed, consisting of binary plasmids encoding RNA1, RNA3, a modified version of RNA5 in which the coding sequence of iLOV or green fluorescent protein (GFP) replaces the RNA5 ORF, and the cucumber mosaic virus (CMV) 2b silencing suppressor. Co-delivery of RNA2 cDNAs, which produce viral glycoproteins, yields virus-like particles (VLPs) (Urrutia et al., 2022; Verchot et al., 2020). A stable full-length infectious clone system for RRV has been used for studying the host range of RRV and systemic accumulation of viral genome segments (Atallah et al., 2022; Verchot et al., 2020). Until now, rosetted shoots have only been observed in naturally infected field plots and not in greenhouse experiments by inoculating rooted cuttings with our infectious clone.

This investigation into the molecular mechanisms underpinning RRD uses an omics-based approach and is made possible by the data mining resources of the Genome Database for Rosaceae (GDR) which provides high quality curated genomes for a large number of species (almond, apple, blackberry, cherry, peach, pear, plum, raspberry, rose, and strawberry) (Jung et al., 2019). Leaves were harvested from the symptomatic rosetted and asymptomatic branches of RRV infected rose plants in a single research field plot, while healthy plants were obtained from another plot. Plants became infected naturally through feeding by viruliferous eriophyid mites, *Phyllocoptes fructiphilus* (Di Bello et al., 2018; Otero-Colina et al., 2018). Transcriptomic datasets were used to identify gene sets that were uniquely altered in symptomatic and asymptomatic tissues of field grown rose plants. This investigation significantly advances our understanding of how RRV impacts the crosstalk among gene networks that tailor growth and development in infected tissues.

## Methods

### Plant material, cDNA library construction, illumina sequencing

Leaf samples from rosetted and asymptomatic branches were collected from ten field-grown rose shrubs (var. Double Knockout) in Waco, Texas USA (lat. 31.56N, long. -97.14W). Ten samples of healthy rose shrubs (var. Double Knockout) were collected from another College Station, Texas (USA) field plot (lat. 30.62 N, long, 96.33W). The shrubs were grown in these plots for more than 3 years. Total RNA extraction was performed using the Maxwell^®^ RSC Plant RNA Kit (Promega Corp. Madison, WI, USA). The ten rosetted, ten asymptomatic, or ten healthy RNAs samples were pooled and then divided into 5 aliquots of 200 ng. Large scale mRNA sequencing was performed using the Illumina NovaSeq platform (Novogene Corp. Sacramento, CA, USA). cDNA library sequencing using a pair-end 150 bp sequencing strategy was used to generate raw data which was initially processed using Fastp to remove adaptors (Chen et al., 2018). FASTQC was used to assess read quality (Andrews, 2015).

### RNA-seq read mapping and transcript level expression analysis

The Texas A&M University High Performance Research Computing services provided the computing environment and resources for this research. HISAT2.2.0 was used to align reads to the reference genome, *Rosa chinensis* Old Blush homozygous genome v2.0 (Kim et al., 2015; Raymond et al., 2018). The SAM files were converted into BAM files using SAMtools (Danecek et al., 2021). Mapped reads were assembled into potential transcripts, and their abundance was estimated using StringTie (Shumate et al., 2022), resulting in the creation of a Ballgown directory (Pertea et al., 2016). Finally, the PrepDE.py tool was used to produce both gene and transcript count files (https://ccb.jhu.edu/software/stringtie/).

Within RStudio, DESeq2 was used for RNA-seq data analysis and for generating volcano plots, Venn diagram, and heat maps (Love et al., 2014; RStudio Team, 2020). We implemented a cut point for differentially regulated genes of ≤ 1 or ≥ 1 log_2_-fold difference and padj <0.05. The DEGs were classified into asymptomatic and symptomatic categories and further subdivided into upregulated or downregulated groups (**Supplementary Table S3 through S10**). The GDR database containing the *Rosa chinensis* Old Blush homozygous genome v2.0 (www.rosaceae.org) and the Gene ontology (GO) knowledgebase were used (https://amigo.geneontology.org/amigo/dd_browse; last accessed 28/07/2024) to obtain gene descriptions and InterPro protein family descriptions. Ensembl Gene IDs were submitted to ShinyGO (http://bioinformatics.sdstate.edu/go/; last accessed 09/16/2024) and DAVID (https://david.ncifcrf.gov/; last accessed 02/10/2024) (Ge et al., 2020; Sherman et al., 2022) to identify significantly enriched biological GO terms with *p <* 0.05. The KEGG database (https://www.kegg.jp/) was used for pathway analysis using the following maps: map00380, map00908, map00906, map00940, and map04075.

### Promoter analysis and protein phylogeny

Promoter sequences (1000 bp upstream from transcription start site) were extracted using the Biomart tool from the Ensembl Plants (https://plants.ensembl.org/; Last accessed 03.03.2028) using the latest *Rosa chinensis* (RchiOBHm-V2) assembly (Dyer et al., 2025; Smedley et al., 2015). *Cis* elements associated with hormone biosynthesis, metabolism, and response were retrieved from the New Place database (https://ngdc.cncb.ac.cn/databasecommons/database/id/5596; Last accessed 01.03.2025) (Higo et al., 1999). These *cis* elements were mapped to the promoters using Geneious Prime ver. 2025.0 (Dotmatics, Boston MA, USA).

Sequences were retrieved from the GDR and The Arabidopsis Information Resource database (TAIR) (Berardini et al., 2015; Jung et al., 2019). PhyloGenes was used to aid selection of appropriate homologues and for phylogenetic tree building and for plant gene functional interference (Zhang et al., 2020b). Multiple sequence analysis and maximum likelihood (ML) phylogenetic trees were generated using Geneious Prime (https://www.geneious.com. The accession IDs for all genes in the phylogenetic analyses are presented in **Supplementary Table S11**. Tree visualization was prepared using iTOL (v5) (Letunic and Bork, 2021).

### Hormone panel and sample preparation

Analysis of metabolites was performed using approximately 100 mg of fresh weight tissue that was placed in 2 mL tubes containing zirconia beads. Samples flash-frozen in liquid nitrogen upon collection and stored at -80°C. Upon extraction, 500 µL of phytohormone extraction buffer (1-propanol/water/HCL[2:1:0.002v/v/v]) containing 500 nM isotopically labeled internal standards [dddABA ([2 H6] (+)-cis,trans-abscisic acid, OlChemIm), dddIAA ([2 H5] indole-3-aceetic acid, OlChemIm), dddJA (2,4,4-d3; acetyl-2,2-d2 JA, CDN Isotopes), and dddSA (d6-SA, Sigma-Aldrich)] was added to each sample. Samples were homogenized and agitated for 30 min. at 4°C, after which 500 µL dichloromethane was added to each tube. Samples were agitated again for 30 min. at 4°C, and then centrifuged at 150000 rpm for 10 min. The lower organic layer was transferred to 2 mL glass vials (Restek Corporation, Bellefonte, PA, USA) and evaporated by N_2_ gas. Dried samples were resuspended in 150 µL LC-grade methanol and transferred to 1.7 mL tubes and placed in -20C for 48 hours. Samples were then filtered with 0.22 µm filters (Sigma-Aldrich, St. Louis, MO). Filtered samples were placed in glass inserts (Restek Corporation, Bellefonte, PA) and placed in 2 mL glass vials and stored at -20°C until run on the LC-MS/MS. A 10 µL aliquot of each sample was injected into a Triple Quad 4500 LC-MS/MS system (Sciex) using electrospray ionization (ESI) in negative ion mode with multiple reaction monitoring (MRM). Chromatography was performed using an Ascentis C18 column (3 cm x 2.1 mm, 2.7 µm; Sigma-Aldrich). The mobile phase gradient was set at a 0.495 mL/min flow rate consisting of solution A (0.02% [v/v] acetic acid in water and solution B (0.02% [v/v] acetic acid in acetonitrile) with the following gradient (time in minutes - % [v/v] solution B): 0.5 – 10%, 1.0 – 20%, 21.0 – 70%, 24.6 – 100%, 24.8 – 10%, 29- stop). All hormones and metabolites were quantified using Analyst v.1.7.2 software by comparison of internal standards.

### RT-PCR analysis of RRV segments and host gene expression

First cDNA was prepared using 150 ng pooled RNA, random hexamer primers, and Maxima™ H Minus Reverse Transcriptase (ThermoFisher Scientific, Waltham, MA, USA). All primers for PCR amplifications are listed in **Supplementary Table S1**. Endpoint RT-PCR was performed using GoTaq^®^ G2 Flex DNA polymerase (Promega Corp.) to detect RRV RNA3 as described previously (Atallah et al., 2022). The qRT-PCR detection of viral RNAs 3, 5, and 6 was performed using 300 nM final concentration of primers and detection of RNA7 was performed using 900 nM final concentration (Dobhal et al., 2016). For qRT-PCR detection of host transcripts we used 500 nM final concentration and the PowerUp™ SYBR™ Green Master Mix (ThermoFisher Scientific, Waltham, MA, USA).

## Results

### Abscisic acid, gibberellin, caffeic acid, and auxin levels in RRV symptomatic and asymptomatic canes

**Figure 1:**
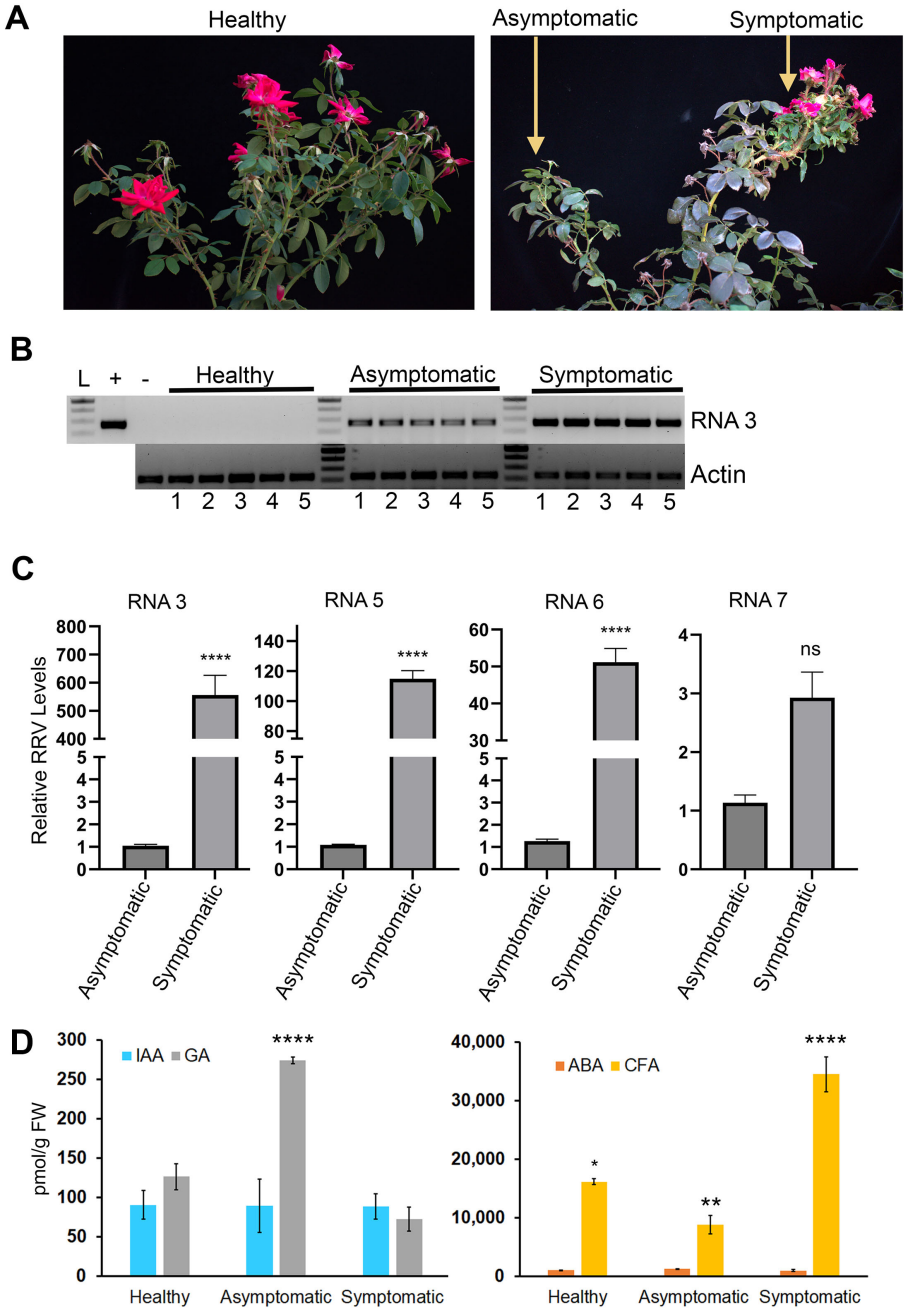
RRV infection along with changes in IAA and ABA in mature field grown plants. **(A)** Representative images plants sampled for RNA sequencing analysis in this study. **(B)** The 271 bp products represent RRV RNA3 obtained by RT-PCR analysis of RNA obtained from plants that were used for RNA-seq analysis. Lane L: 100 bp DNA ladder. The “**+”** identifies a positive control PCR amplification using pCB301-RRV-RNA3 plasmid. The **“-”** identifies RT-PCR products derived from heterologous RNA obtained from greenhouse grown healthy roses used for RT-PCR as a control against the field grown samples. RT-PCR was performed using actin primers as the endogenous control **(C)** Bar charts illustration for qRT-PCR, the relative accumulation of RRV RNA 3, RNA 5, RNA 6 and RNA 7 in symptomatic tissues compared to asymptomatic tissues. ****: Significance level, *p*< 0.0001, “ns”: not significant. **(D)** Average pmol/gFW of IAA (gray bars), GA1 (blue bars), ABA (orange bars), and CFA (gold bars) were comparable between healthy, asymptomatic, and symptomatic tissues (*p*> 0.05). * , **, *** identifies signficiantly different from each other.

To confirm RRV infection in garden rose populations that were clearly displaying rosetting symptoms, endpoint RT-PCR was performed using standard diagnostic primers detecting RNA3 (**Figure 1A**; **Supplementary Table S1**) (Di Bello et al., 2015). The band intensities of the symptomatic samples were stronger than those of the asymptomatic samples (**Figure 1B**). The comparative ddCT method was used to compare the proportion of viral-associated RNAs in symptomatic and asymptomatic tissues. There was approximately 550-fold greater RRV RNA3, 115-fold greater RNA5, 50-fold greater RNA6, and 3-fold greater RNA7 in symptomatic than in asymptomatic samples (**Figure 1C**; *p* **<**.0.0001). The relative concentrations of genome segments and corresponding mRNAs vary significantly among the canes and shoots of the host plant in a manner that positively correlates with the display of RRD (Gallet et al., 2022, 2018).

To test the hypothesis that unusual levels of growth promoting hormones might be an underlying cause for this disease, we measure ABA, caffeic acid (CFA), gibberellin 1 (GA), and indole-3-acetic acid (IAA) levels in healthy, asymptomatic infected, and symptomatic rosetting infected tissues. Measures of zeatin, salicylic acid, and jasmonic acid are reserved for a related study. CFA levels were elevated in symptomatic tissues, while GA levels were elevated in asymptomatic tissues. Our analysis failed to identify statistical differences for ABA and IAA among infected and healthy tissues (**Figure 1D**; *p*<0.05). A controlled greenhouse study in which we compare mechanical virus inoculation, viruliferous mite infestations, and aviruliferous mite infestations and collect various tissue samples at precise times prior to and including the onset of rosetting disease is needed to assess potential differences. However, such mite populations are not yet available for this type of work.

### Differentially expressed genes in asymptomatic and symptomatic leaves

We hypothesized that RRD is the result of critically altered changes in host gene expression, which can be controlled by plant hormones. In order to understand the genetic re-programming that underlies rosette disease, we compared the transcriptomic responses between symptomatic and asymptomatic tissues. High-throughput RNA sequencing generated a total of 681,907,397 paired reads (**Supplementary Figure S1A**). The overall average mapping percentage was 86% across all samples, which is favorable for downstream scrutiny of gene expression between different conditions (**Supplementary Table S2**). An overall total of 509,183,333 paired reads aligned to the reference genome, with 167,237,081 from asymptomatic samples, 176,719,842 from symptomatic samples, and 165,226,410 from healthy samples. A total of 155,223,705 paired reads were uniquely mapped to the reference genome, including 49,346,142 from asymptomatic samples, 50,039,016 from symptomatic samples, and 55,838,547 from healthy samples. Additionally, 17,298,359 paired reads were multi-mapped to the reference genome, comprising 5,183,163 from asymptomatic samples, 6,061,818 from symptomatic samples, and 6,053,378 from healthy samples. The statistical reliability of the data was assessed using dispersion plot analysis (**Supplementary Figure S1B**). The total number of differentially expressed genes was obtained using DeSeq2, while omitting low abundance genes (<10 counts, padj <0.05).

**Figure 2:**
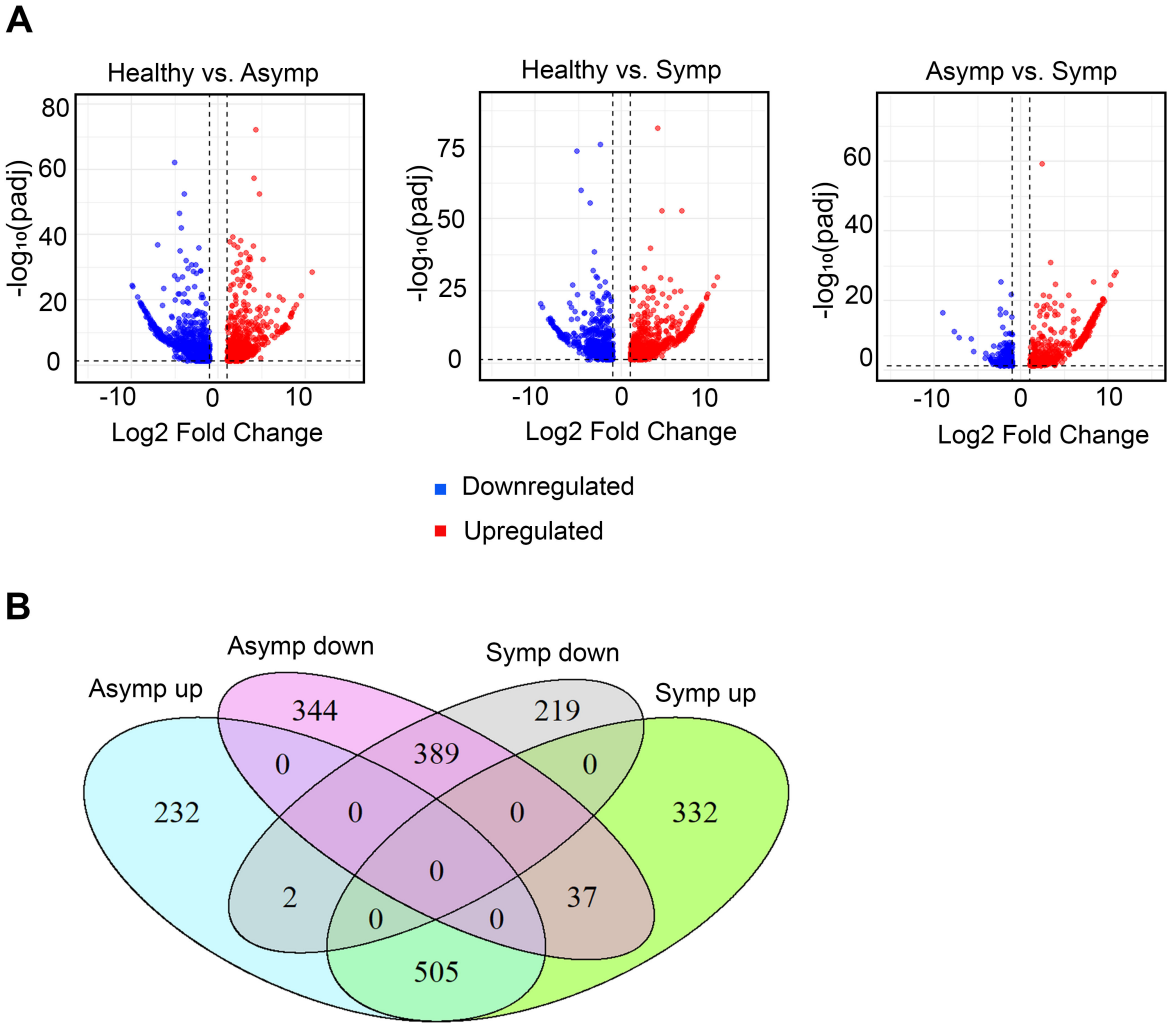
Differentially expressed genes (DEGs) in asymptomatic and symptomatic tissues. **(A)** Volcano plots showing the contrasting patterns of gene expression between asymptomatic and symptomatic tissues compared to healthy respectively as well as symptomatic compared to asymptomatic tissues. The X-axis represents the magnifold change in gene expression and the Y-axis represents the statistical significance of change (-log_10_(padj)). Blue and red dots represent downregulated and upregulated genes, respectively. **(B)** Venn diagram representing shared and unique DEGs in asymptomatic and symptomatic tissues.

The log2-fold changes in up- and down- regulated genes in the asymptomatic compared to healthy and symptomatic compared to healthy leaves were similarly distributed. However, when we compare the asymptomatic to the symptomatic datasets there were more upregulated gene (**Figure 2A**). The total number of differentially expressed genes (DEGs) in asymptomatic and symptomatic compared to healthy leaves were 1509 and 1484, respectively (**Table 1**). A total of 739 genes were upregulated in asymptomatic and 874 were upregulated in the symptomatic tissues, while 770 and 610 were downregulated in asymptomatic and symptomatic tissues, respectively (**Table 1**). A Venn diagram presents the DEG populations that were commonly or uniquely expressed among the asymptomatic and symptomatic datasets (**Figure 2B**). There were 389 commonly downregulated and 505 commonly upregulated genes in both asymptomatic and symptomatic tissues. Respectively in asymptomatic and symptomatic tissues, 219 and 344 genes were uniquely downregulated, 232 and 332 genes were uniquely upregulated, and 37 genes were downregulated in asymptomatic while oppositely regulated in symptomatic (**Figure 2B**; **Supplementary Tables S3**, **S9**). Two genes encoding a putative polygalacturonase (Chr5g0021031) and another whose function is unknown (Chr2g0123501) were upregulated in asymptomatic tissues and oppositely regulated in symptomatic samples. Polygalacturonase is a hydrolytic enzyme that is not central to virus-host interactions per se (**Supplementary Tables S9**, **S10**). The GDR database gene description or InterPro classification of functional domains (Blum et al., 2025) for these 37 plus 2 genes did not point to any obvious developmental gene networks that could be used to directly infer specific molecular mechanisms were subverted to cause rosetting disease.

**Table 1:** Number of differentially expressed genes RRV infected plants.

Comparison of treatments	Total no. regulated genes	Total no. upregulated genes	Total no. downregulated genes
Asymptomatic vs. Healthy	1509	739	770
Symptomatic vs. Healthy	1484	874	610

### Gene expression associated with plant organ and flower development were intensified in rosetted tissues

**Figure 3:**
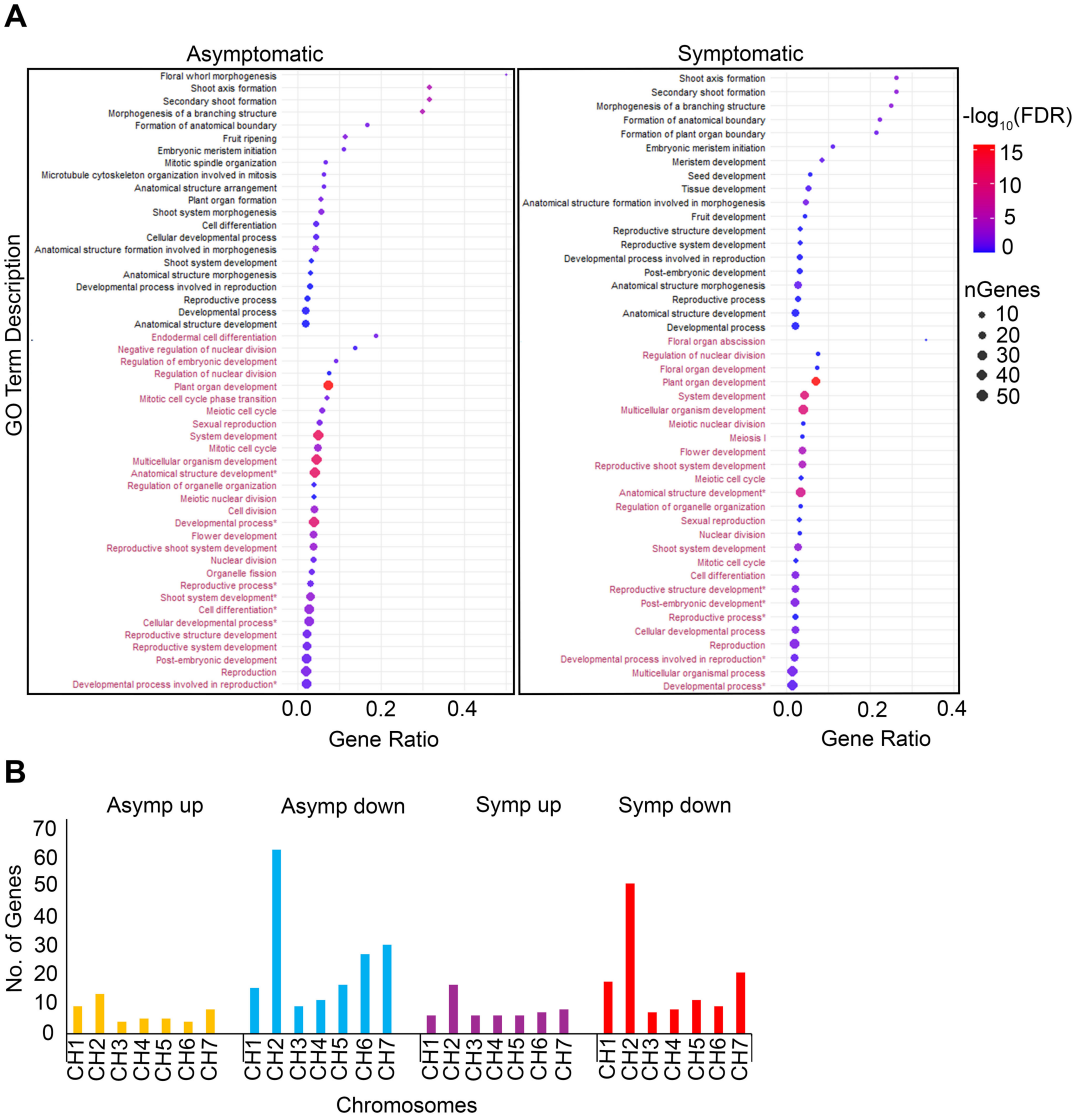
Developmental GO terms enrichments and chromosomal distribution of genes. **(A)** Dot plots are comprised of 50 (left) and 45 (right) GO terms in asymptomatic and symptomatic tissues respectively. GO terms colored black represent upregulated GO terms and maroon represent downregulated GO terms. Gene ratio represents the proportion of genes relative to the total number of genes affiliated with the GO term. Dot size corresponds to the number of genes associated with the GO term. Dots color represent -log10(FDR), indicating significance. **(B)** Bar chart showing the chromosomal distribution of genes represented in the dot plots. Y – axis; total number of genes count. X – axis; chromosomes distribution.

Gene classes affiliated with plant development and morphology were identified using GO enrichment of terms associated with biological processes (**Figure 3A**). These data highlighted significant changes in gene expression relating to plant growth and development while also suggesting that rosetting disease may be linked to more subtle genetic changes. For example, three terms, ‘Shoot axis formation’, ‘Secondary shoot formation’, and ‘Morphogenesis of a branching structure’ had higher gene ratios and FDR values in asymptomatic (gene ratio > 0.3; -log_10_ FDR > 5) than symptomatic tissues (gene ratio > 0.25; -log_10_ FDR <5) which signifies that rosetting disease resulted from defined changes in gene expression. GO terms with the largest number of genes and high FDRs (-log_10_ FDR range 10-15) in asymptomatic and symptomatic tissues included ‘Plant organ development’, ‘System development’, ‘Multicellular organism development’, and ‘Anatomical structure development’. The downregulated GO terms ‘Organelle fission’, ‘Endodermal cell differentiation’ and ‘Negative regulation of nuclear cell division’ were unique in the asymptomatic dataset. ‘Nuclear division’, ‘Mitosis cell cycle’, and ‘Meiotic cell cycle’ have higher FDR values in asymptomatic (-log_10_ FDR >5) than symptomatic (-log_10_ FDR > 0 and less than 0.001) tissues. Terms that were uniquely upregulated in symptomatic tissues included ‘Formation of plant organ boundary’, ‘Seed development’, ‘Tissue development’, ‘Fruit development’, and ‘Reproductive structure/system development’. These five unique terms support our hypothesis that rosette disease includes factors functioning at the shoot apex and in reproductive development (**Figure 3A**).

Notably, upregulated genes in symptomatic and asymptomatic tissues were similarly distributed across all host chromosomes, with more than 10 genes associated with chromosome 2 (**Figure 3B**). There were also more downregulated genes associated with chromosome 5, 6, and 7 in asymptomatic than symptomatic tissues. Between 45 and 70 downregulated genes were associated with chromosome 2 in asymptomatic and symptomatic tissues. The downregulated genes both asymptomatic and symptomatic datasets located along chromosomes 2 and 7 encode 32 WOX-family transcription factors, DNA polymerases, ribonuclease H/reverse transcriptase, RNA helicases putatively involved in the maturation of SSU-rRNA, and many uncharacterized factors (**Supplementary Tables S3**, **S6**). Given that WOX-family genes are primarily involved in shoot growth, apical and lateral meristem development, it was surprising that so many were downregulated in asymptomatic tissues which appear to produce normal branches, leaves, and flowers (Costanzo et al., 2014; Deveaux et al., 2008; Rasheed et al., 2024).

### Analysis of DEGs linked to hormone biosynthesis, catabolism, and signaling involved in vegetative and reproductive development

**Figure 4:**
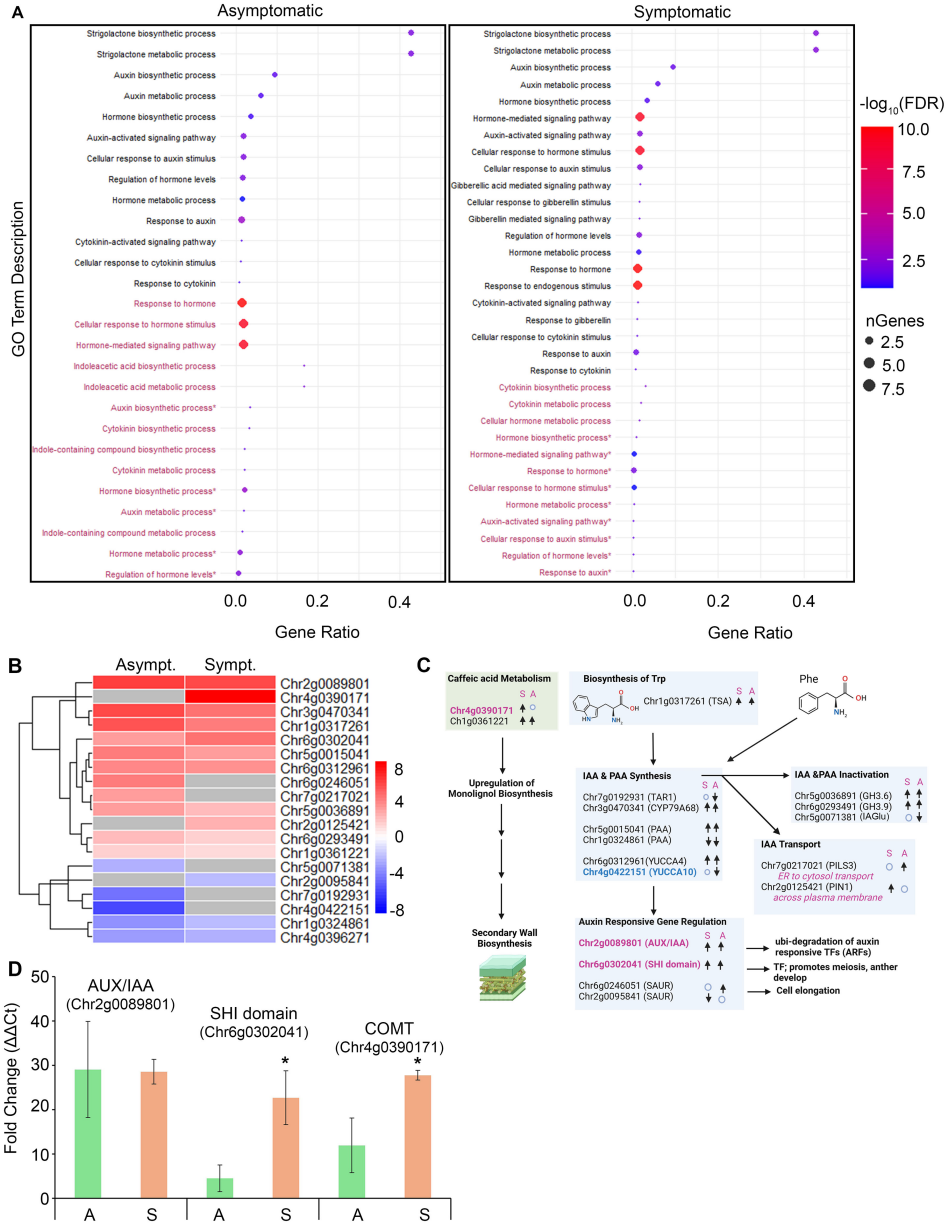
Developmental hormone GO term enrichment and gene expression analysis in infected tissues. **(A)** Dot plots representing 27 (left) and 33 (right) GO terms in asymptomatic and symptomatic tissue respectively. Black colored GO terms represent upregulated GO terms and maroon represent downregulated GO terms. Gene ratio represents the proportion of genes relative to the total number of genes affiliated with the GO term. Dot size corresponds to the number of genes associated with the GO term. Dots color represents -log10(FDR), indicating significance. **(B)** Heatmap displaying auxin related DEGs in symptomatic and asymptomatic tissues. Rows represent individual genes. Color scale indicates normalized expression (log_2_ Fold Change), red representing upregulation and blue representing downregulation. **(C)** Schematic representation of genes involved in caffeic acid and auxin biosynthesis, metabolism, and signaling. “↑”, “↓” represents upregulated and downregulated gene expression respectively. “o” indicates no change in gene expression. S, Symptomatic tissues; A, Asymptomatic tissues. **(D)** Bar chart showing relative gene expression levels of auxin related genes in symptomatic and asymptomatic tissues. Green bars represent asymptomatic tissues, light brown bars represent symptomatic tissues. * indicates significant difference between symptomatic tissues and asymptomatic tissues in paired analysis.

GO term enrichment for phytohormone metabolic and response pathways revealed generally low gene ratios (<0.025), except for the metabolic and biosynthetic processes of SLs and auxin (**Figure 4A**). The gene numbers, ratios, and FDR values for SL biosynthesis and metabolism were upregulated in both symptomatic and asymptomatic tissues (gene ratio >0.4; -log10FDR >4). SLs regulate lateral bud development and branch development by influencing polar auxin transport and cytokinin levels (Brewer et al., 2009; Zha et al., 2022). Other broad terms, ‘Response to hormones’, ‘Cellular response to hormone stimulus’ and ‘Hormone-mediated signaling pathway’ (~10 genes per term) in asymptomatic tissues were downregulated and oppositely regulated in symptomatic tissues (gene ratio <0.025; -log FDR ~10.0) (**Figure 4A**). This supports our hypothesis that unknown changes in hormone activities contribute to the manifestation of rosetting disease.

A heatmap was used to display the differential expression patterns of nineteen genes linked to auxin biosynthesis from tryptophan or phenylalanine, catabolism, transport, signaling, and auxin responsive genes (**Figures 4B, C**). These genes belong to large well-characterized families that contribute to apical and axillary bud growth, flowering, shoot branching, and vascular development (Cheng et al., 2006; Estornell et al., 2018; He et al., 2018; Zhang et al., 2022). Regarding auxin biosynthesis, seven DEGs were involved in the synthesis of indole 3-acetic acid (IAA) and phenylacetic acid (PAA) (**Figures 4B, C**) (Zhang et al., 2022). Chr6g0312961 and Chr4g0422151 encoded genes identified as YUCCA4 and YUCCA10 respectively, which encode indole-3-pyruvate mono-oxygenases. (**Supplementary Tables S3**-**S5**, **S7**). YUCCA4 and YUCCA10/11 monooxygenases catalyze rate-limiting steps in the synthesis of IAA and PAA. We performed sequence alignments and maximum-likelihood phylogenetic analysis (PHYML) of *YUCCA* family genes from almond, apple, Arabidopsis, tomato, roses, and strawberry to confirm that these genes group with *YUCCA4* and *YUCCA10/11* clades (**Supplementary Figure S2**). The *RcYUCCA10* was 8-fold downregulated in asymptomatic tissues. In symptomatic tissues, *RcYUCCA10* was not significantly altered, suggesting that the altered balance of *RcYUCCA4*:*RcYUCCA10* expression might contribute to abnormal floral development (Cheng et al., 2006).

*AUX/IAA* (Auxin/Indole-3-acetic acid), *SHI_STY* (SHORT INTERNODES/STYLISH), and *SAUR* (Small Auxin-Upregulated RNAs) encode auxin signaling factors that influence plant stature, leaf and flower development, as well as stress responses. Their dysregulation by overexpression or knockout can cause developmental abnormalities in plants (Bao et al., 2024). SHI/STY transcription factors positively regulate *YUCCA* gene expression by binding at cis elements in their promoters (Bao et al., 2024; Estornell et al., 2018; Powers and Strader, 2020). Transcripts encoded by Chr2g0089801 and Chr6g0302041 encode AUX/IAA-domain and SHI-domain proteins respectively, and were upregulated in both asymptomatic and symptomatic datasets (**Figure 4B**). Transcript levels associated with Chr6g0246051 and Chr2g0095841, which encode SAUR-domain proteins and were alternatively up and downregulated in infected tissues (**Figures 4B, C**). SAUR proteins are known to induce cell elongation and growth, thus the downregulation of *SAUR* mRNA in symptomatic tissues might have influenced rosetting (van Mourik et al., 2017).

Polar auxin transport is crucial for regulating cell elongation, apical and axillary bud development, vascular development, and root growth. PIN-FORMED (PIN) protein family of auxin exporters have a long internal hydrophilic loop and are distributed at the plasma membrane for directional transport of auxin out of the cell. PIN-LIKES proteins (PILS) are structurally similar to PINS but have short internal hydrophilic loops, reside at the endoplasmic reticulum (ER), and controls nuclear abundance and signaling of auxins. PIN and PILS work together to control important determinants of plant architecture (Estornell et al., 2018; Sauer and Kleine-Vehn, 2019; Zhang et al., 2022). Transcripts encoded by Chr2g0125421 and Chr7g0217021, respectively, encode probably PIN1 and PILS3 proteins and were upregulated in RRV infected tissues (**Figure 4B**). Sequence alignments and maximum-likelihood phylogenetic analysis (PHYML) of *PIN* and *PILS* family genes from almond, apple, Arabidopsis, tomato, roses and strawberry were performed, confirming the accuracy of the gene descriptions provided in the GDR database (**Supplementary Figure S3**). The Chr2g0125421 associated PIN1 groups in a subclade with strawberry PIN1 and PIN2 proteins. Transcripts of Chr7g0217021 encodes a protein that clusters with PILS3 of strawberry. Roses encode seven PILS1/3 genes while almond, apple, and strawberry encode three or four PILS1/3 genes (**Supplementary Figure S3**). The preferential upregulation of *PINS* mRNA in symptomatic tissue implies that auxin depletion might have a greater contribution to rosetting disease than the increase in nuclear auxin abundance caused by heightened *PILS* mRNA expression in asymptomatic tissue (Béziat et al., 2017; Feraru et al., 2019; Martinez et al., 2016; Sun et al., 2020; Zhou and Luo, 2018).

Class II *GH 3* (*GRETCHEN HAGEN 3*) genes encode auxin-amido synthetases which promote the inactivation of IAA by conjugation with amino acids. *GH3*-dependent IAA conjugation can alter the ratio of IAA and PAA in growing plants and significantly influence leaf and root development in response to biotic and abiotic stimuli (Aoi et al., 2020; Guo et al., 2022; Luo et al., 2023). *GH3.6* and *GH3.9* factors were upregulated in response to RRV infection in symptomatic and asymptomatic tissues (**Figures 4B, C**). Indole-3-acetate beta-glucosyltransferase (IAGlu synthase) conjugates IAA with glucose and is best studied in monocot seeds (He et al., 2020). Downregulation of IAA-glucose in virus-infected plants suggests that free auxins, glucose-conjugated IAAs, or IAA conjugated to amino acids were preferred during plant-virus interactions (**Figures 4B, C**).

CFA is sometimes considered an auxin-like factor, influencing shoot growth. We identified two genes encoding putative caffeic acid 3-O-methyltransferases (COMT), key players in lignin biosynthesis for secondary cell walls, xylem and tubular cells (Gao et al., 2024; Liu et al., 2018). Transcripts encoded by a gene at the Chr4g0390171 location is upregulated 8-fold upregulated in symptomatic tissues compared to healthy tissues and was absent from the asymptomatic dataset, while mRNAs of Chr1g0361221 showed mildly upregulation in both datasets (**Figures 4B, C**). Upregulation of both CFA and lignin biosynthesis in symptomatic tissues might contribute to thickening shoots and prickle development.

Next, we selected three genes for RT-qPCR to compare their expression levels in asymptomatic and symptomatic tissues that we collected at a later time from the same plants used for RNA-seq analysis to assess the reproducibility of this earlier analysis. RT-qPCR analysis of Chr2g0089801, Chr6g0302041, and Chr4g0390171 encoded transcripts confirmed their significant upregulation in symptomatic tissues, consistent with the RNA-seq data (**Figure 4D**, p *<*0.05). The *COMT* gene, which showed no induction in asymptomatic tissues by RNA-seq, was somewhat induced in these tissues by RT-qPCR. Comparing outcomes of RNA-seq and RT-qPCR, both datasets revealed significant and consistent changes in gene expression levels. Thus, comparing asymptomatic and symptomatic tissues collected at different times, we can assert that the level of gene expression changes likely co-varies dynamically in field grown plants (**Figure 4D**).

### Differential gene expression shaping IAA and CFA metabolism and signaling

Expression of auxin biosynthetic and signaling genes is well known to be regulated by direct feedback and by crosstalk with other plant hormones. In the above ground parts of the plant, crosstalk between IAA, CKs, and GAs contributes to shoot development, cell elongation, and vascular development, as organ boundaries and leaf patterning (Hepworth et al., 2005; Qiu et al., 2019; Schäfer et al., 2015). We analyzed *cis-*elements in the promoters of the 18 genes provided in **Figure 4** and identified between four and nineteen GA-responsive elements (**Table 2**), surpassing the number of other hormone responsive elements in the same promoters (Bouré and Arnaud, 2023). These data suggest that GA is more likely plays a pivotal role in rosetting disease. Cytokinin responsive elements were either absent or occur in low numbers. ABA and auxin-responsive elements were the second most highly represented (**Table 2**). Overall, these data suggest that rosetting disease is greatly influenced by crosstalk between CK, GA, auxin, and ABA.

**Table 2:** Promoter analysis for auxin related responsive genes.

Locus ID	CK	ABA	Auxin	GA	Description
Chr5g0071381	0	7	4	19	Indole-3-acetate beta-glucosyltransferase
Chr2g0089801	0	1	3	16	Auxin-responsive protein IAA1
Chr7g0217021	0	3	3	8	Auxin Transport (PIN-efflux)
Chr2g0125421	0	1	2	4	Auxin Transport (PIN-efflux)
Chr1g0324861	2	1	4	8	Phenylacetaldehyde synthase
Chr7g0192931	1	0	4	9	Tryptophan aminotransferase-related protein 1
Chr1g0317261	3	2	7	10	Tryptophan synthase alpha chain
Chr5g0015041	3	4	2	10	Phenylacetaldehyde synthase
Chr3g0470341	0	3	5	16	CYP79A68
Chr4g0422151	2	5	5	9	YUCCA10/11-like
Chr6g0312961	2	3	1	8	YUCCA4-like
Chr5g0036891	0	1	1	8	Indole-3-acetic acid-amido synthetase GH3.6
Chr6g0293491	0	1	3	5	Indole-3-acetic acid-amido synthetase GH3.9
Chr2g0095841	1	0	4	9	SAUR family
Chr6g0246051	2	3	4	13	SAUR family
Chr6g0302041	0	4	2	7	SHI related sequence 1
Chr1g0361221	1	3	2	12	Caffeic acid 3-O-methyltransferase (COMT)
Chr4g0390171	2	0	0	10	Caffeic acid 3-O-methyltransferase (COMT)

### DEGs linked to ABA, SL, GA, and zeatin biosynthesis have a contrasting influence on rosetting disease

**Figure 5:**
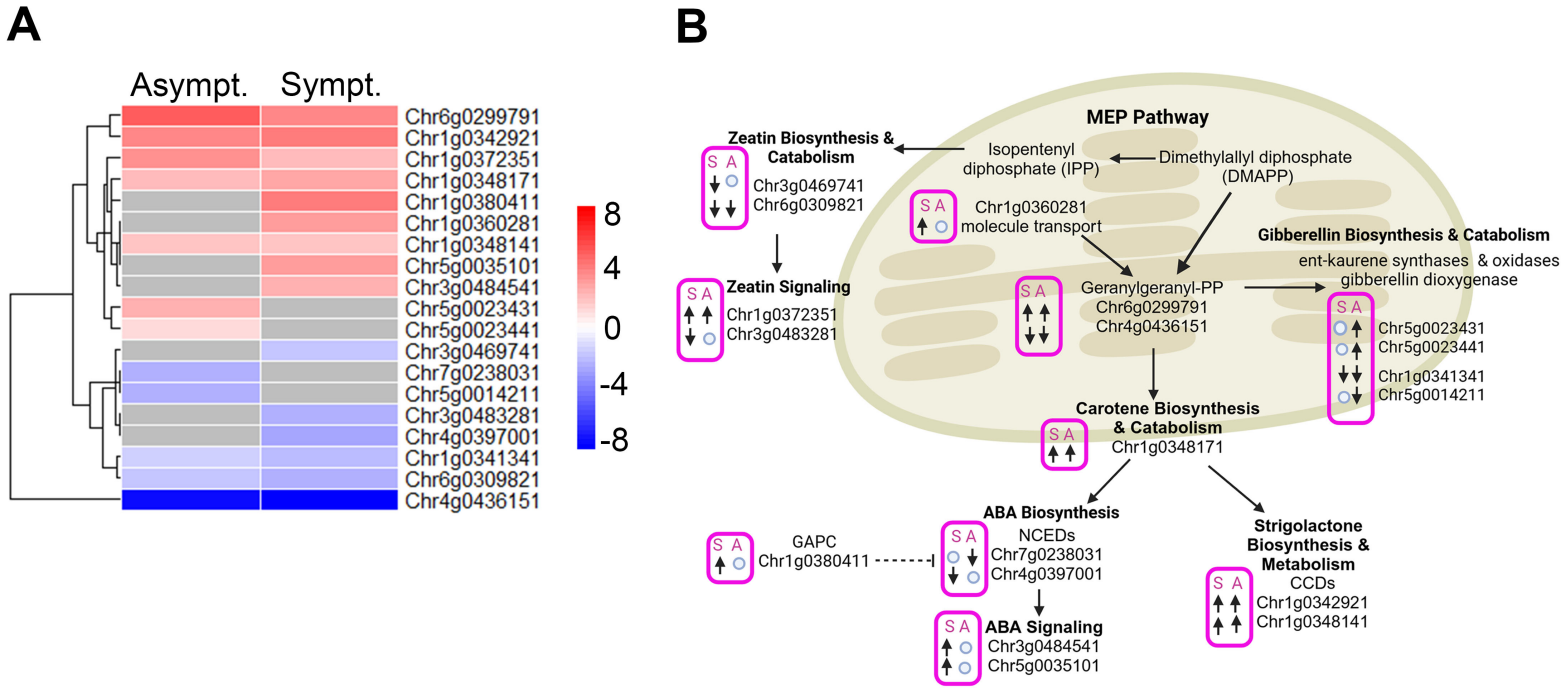
DEGs associated with SLs, ABA, CK and GA biosynthesis, metabolism and signaling in infected tissues. **(A)** Heatmap displaying the expression levels of DEGs associated with SLs, ABA, CK and GA biosynthesis, metabolism and signaling in symptomatic and asymptomatic tissues. Rows represent genes. Color scale indicates normalized expression (log_2_ Fold Change), red representing upregulation and blue representing downregulation. **(B)** A schematic model highlighting the biological pathways associated with SLs, ABA, CK and GA biosynthesis, metabolism and signaling in symptomatic and asymptomatic tissues in panel **(A)** “↑”; represent upregulated genes, “↓”; represent downregulated genes and ○: no change in gene expression. S, Symptomatic tissues and A, Asymptomatic tissues.

The methyl erythritol phosphate (MEP) pathway is responsible for the biosynthesis of isopentenyl pyrophosphate (IPP) and dimethylallyl pyrophosphate (DMAPP), which are essential precursors to geranylgeranyl diphosphate (GGPP), which is upstream of ABA, SL, and GA biosynthesis. One branch of IPP leads to the synthesis of the CK known as zeatin. We identified 19 DEGs linked to the synthesis, metabolism, and signaling of these hormones (**Figure 5**). A total of 11 genes were upregulated and 8 downregulated in asymptomatic and symptomatic tissues, respectively.

Zeatin is a cytokinin that promotes cell division, lateral bud development, and shoot growth. Transcripts associated with Chr3g0469741 and Chr6g0309821 encode putative zeatin biosynthetic proteins (LOG3 and SOB5-like), and activation were downregulated in infected tissues (**Figure 5**). Chr1g0372351 encodes the putative *ARR-17* (type-A response regulator17-like factor), which suppresses CK signaling and was upregulated in infected tissues. Chr3g0483281 encodes a putative *ZOG* (zeatin O-glycosyltransferase) which is involved in the inactivation and storage of zeatin and was downregulated in symptomatic tissues and unaffected in asymptomatic tissues (**Figure 5**). The reduction in synthesis, activation, and storage of zeatin, suggests that CKs are important drivers in the appearance of rosetting disease. Examining the phytohormone regulated *cis*-elements in the promoters for the four zeatin metabolic genes presented in **Figure 5**, their expression or repression is controlled mostly by GA and to a lesser extent by auxin (**Table 3**).

**Table 3:** Promoter analysis for CK, ABA, GA and SLs related responsive genes.

Locus ID	CK	ABA	Auxin	GA	Description
Chr1g0360281	0	0	4	8	Isopentenyl diphosphate biosynthetic process
Chr6g0299791	0	4	4	15	Homogentisate geranylgeranyl transferase
Chr4g0436151	1	1	1	8	Geranyl diphosphate phosphohydrolase-like
Chr3g0469741	1	2	7	6	Cytokinin riboside 5’-monophosphate phosphoribohydrolase LOG3
Chr3g0483281	0	1	1	19	Zeatin O-glucosyltransferase
Chr6g0309821	0	0	3	7	SOB FIVE-LIKE 5; Cytokinin biosynthesis/signaling
Chr1g0372351	2	1	2	12	Two-component response regulator ARR17
Chr1g0348171	0	4	1	14	Carotene catabolic process
Chr1g0348141	0	3	2	16	Carotenoid cleavage dioxygenase 7
Chr1g0342921	0	3	5	9	Carotenoid cleavage dioxygenase 7
Chr7g0238031	1	4	1	11	9-cis-epoxycarotenoid dioxygenase NCED2
Chr4g0397001	1	0	2	8	9-cis-epoxycarotenoid dioxygenase NCED6
Chr1g0380411	2	2	8	10	Glyceraldehyde-3-phosphate dehydrogenase (GAPC)
Chr1g0341341	0	2	3	17	Ent-kaurenoic acid oxidase 1
Chr5g0023431	0	2	3	10	Ent-kaurene synthase
Chr5g0023441	0	6	5	12	Ent-kaurene synthase
Chr5g0014211	1	0	2	7	Gibberellin 2-beta-dioxygenase
Chr3g0484541	1	0	3	11	ABA responsive glycosyltransferase-like
Chr5g0035101	0	3	1	6	ABA signaling

Seven genes controlling geranylgeranyl diphosphate (GGPP) and GA metabolism were up and downregulated (**Supplementary Tables S3**-**10**). Four GA biosynthetic and catabolic genes were more affected in asymptomatic than symptomatic tissues (**Figure 5**). These genes’ promoters contain numerous GA-responsive elements and fewer ABA or auxin elements. ABA and SL derive from the β-carotene pathway (Czarnecki et al., 2013) Chr4g0397001 and Chr7g0238031 encode putative *NCED2* (9-cis-epoxycarotenoid dioxygenases) and *NCED6*, which control rate-limiting steps in ABA biosynthesis, respectively. The reduced transcript levels may be linked to the heightened levels of a putative cytosolic GAPC (glyceraldehyde-3-phosphate dehydrogenase) that functions as a suppressor of ABA biosynthesis. Their promoters also have an abundance of GA regulated *cis* elements, and the putative *NCED2* has four ABA regulatory elements, which may also contribute to the differential suppression of these genes (**Figure 5**; **Table 3**). Thus, upregulation of mRNAs encoding two ABA signaling factors in symptomatic tissue is not accompanying changes in ABA biosynthesis: a) Chr3g0484541 is linked to a putative ABA....... b) Chr5g0035101 is linked to a putative ABA-binding protein (**Supplementary Table S5**), which inhibits phosphatases that could inactivate kinases involved in ABA-signaling. The dramatic changes in ABA related gene expression does not appear to result in an increase in the pool of bioactive ABA. The transcripts encoding the NCED2 protein and an ABA-binding protein have more ABA-responsive elements than other genes, suggesting there is feedback regulation of these genes, although with less influence than GA (**Table 3**).

Two genes attributed to Chr1g0342921 and CHr1g0348141, encoded probable carotenoid cleavage dioxygenases involved in SL biosynthesis, identified as Chr1g, and were upregulated in symptomatic and asymptomatic tissues. Very little is known about SL's role in plant development and gene expression, although reports link SLs to the inhibition of PIN-auxin transport (Zhang et al., 2020a) and cytokinin antagonism (Duan et al., 2019). SL-responsive promoter elements are not known. These combined data suggest that GA and SLs have significant roles in regulating ABA and zeatin in RRV infected plants.

### Differentially expressed factors influencing organ formation, organ boundary specification, and prickle development

**Figure 6:**
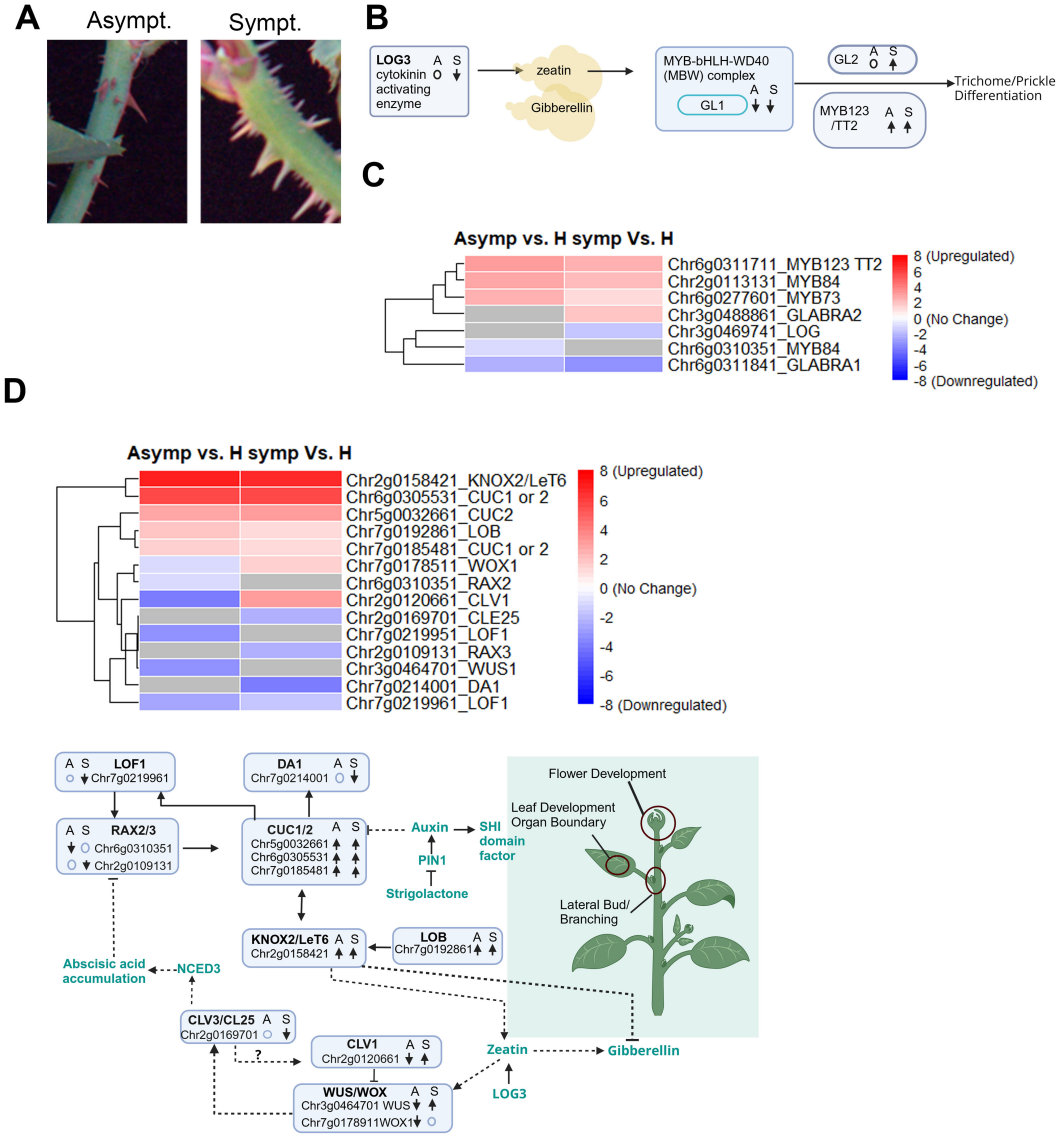
DEGs involved in maintenance of flower, leaf and lateral meristematic cells and prickle developments. **(A)** Representative images showing phenotype difference between asymptomatic (left) and symptomatic (right) prickle/thorns. **(B)** Model summarizing the DEGs altered in symptomatic and asymptomatic tissues influencing phenotype difference in prickles/thorns. “↑”, “↓” represents upregulated and downregulated gene respectively. “o” indicates no change in gene expression. S: Symptomatic tissues, A: Asymptomatic tissues. **(C)** Heatmap displaying DEGs expression levels in symptomatic and asymptomatic tissues involved in prickle/thorns developments. Rows represent genes. Color scale indicates normalized expression (log_2_ Fold Change), with red representing upregulation and blue representing downregulation. **(D)** Heatmap displaying DEGs involved in flower, leaf and lateral bud developments in symptomatic and asymptomatic tissues. Rows represent genes. Color scale indicates normalized expression (log_2_ Fold Change), with red representing upregulation and blue representing downregulation. **(E)** Conceptual representation of genes and transcription factors interaction in symptomatic and asymptomatic tissues to bring phenotype differences in flower, leaf and lateral bud developments. “↑”; represent upregulated genes, “↓”; represent downregulated genes, ○: no change in gene expression. S, Symptomatic tissues; A, Asymptomatic tissues.

Rosetted shoots have an abundance of prickles compared to asymptomatic or healthy branches. These prickles were larger; have white, green, or brown coloration; and were oriented perpendicular to the stem. Prickles in healthy or asymptomatic tissues were smaller, have red or green coloration, and were oriented at a downward angle (**Figure 6A**). Mapping studies identified a core set of prickle regulatory factors that are conserved across vascular plants (Satterlee et al., 2024; Swarnkar et al., 2021), which includes the Lonely Guy (*LOG*) family of phosphoribohydrolases that are essential to produce active CKs (Chen et al., 2022; Kuroha et al., 2009). Spatiotemporal expression patterns of *LOG*s combined with long distance mobility of CKs control cell division and cell expansion needed for leaf development, phyllotaxis patterning, stem prickles, as well as the maintenance of shoot apical and lateral meristems (Wu et al., 2021). *LOG* mutations cause abnormal branching, leaves, and flowers, and loss of prickles (Brandizzi, 2019; Chen et al., 2022; Jones et al., 2010). In our dataset, a single *LOG3* gene was downregulated in symptomatic tissue and unaffected in asymptomatic tissues. This considerable reduction *LOG* gene expression may be an underlying cause of the abnormal growth patterns in rosetted shoots. However, *LOG3* downregulation cannot explain the abundance of prickles in rosetted tissues (**Figures 6A–C**).

Another set of regulators controlling prickle development as well as phenylpropanoid accumulation is the MYB-bHLH-WD40 (MBW) activator-inhibitor complex, which includes R2R3-MYB, bHLH (basic helix-loop-helix) and WDR (WD40 repeat) transcription factors (**Figure 6B**) (Han et al., 2019; He et al., 2023; Jing et al., 2023; Rajput et al., 2022; Shi et al., 2024). A MBW complex that includes *GLABRA 1*(*GL1*) induces *GLABRA2* which is involved in cell fate determination and proanthocyanidin accumulation (**Figure 6C**) (Chen and Wang, 2019; Liu et al., 2024; Pattanaik et al., 2014). In our dataset, *GL1* was conspicuously downregulated while *MYB123-TT2* were upregulated in RRV infected tissues. Surprisingly *GL2* was only found to be upregulated in symptomatic tissues, suggesting that other factors may be influencing *GL2* expression in asymptomatic tissues (**Figure 6C**). Swarnkar et al. (2021) identified additional MYB factors that were differentially regulated during prickle development and three of these were found among the DEGs in RRV infected leaves. Transcripts encoded by Chr6g0277601 (*MYB73*) and Chr2g0113131 (*MYB84*) were upregulated in RRV infected tissues, while Chr6g0310351 (*MYB84*) was specifically downregulated in asymptomatic tissues (**Supplementary Tables S3**, **S4**; **Figure 6C**). These data show that RRV specifically alters the gene regulatory network that controls cytokinin influences on leaf and prickle development.

Another principal gene regulatory network led by *CUC* (*CUP SHAPED COTYLEDON*) and *KNOX* (*KNOTTED1-like homeobox*) influences apical and lateral meristem maintenance, flower and leaf development, were upregulated by RRV infection. LOB (Lateral Organ Boundary) is a transcription factors are proteins that controls expression of the *KNOX2-LeT6* and both mRNAs were upregulated in RRV infected leaves. Since the transcript levels were similar in asymptomatic and symptomatic tissues, it is not likely that these factors drive rosetting disease in these plants. The LOF1 and RAX2/RAX3 genes encode transcription factors that can stimulate CUC expression (Maugarny et al., 2016; Spinelli et al., 2011) were downregulated in RRV infected leaves. The CUC-DA1 regulatory module controls the development of lateral branches (Cui et al., 2024). Given that *DA1* mRNA is downregulated only in symptomatic leaves, its expression may be influenced by factors other than these three *CUC* genes during RRV infection.

The CLV-WUS (CLAVATA-WUSCHEL) feedback signaling module is vital to the maintenance of undifferentiated meristems, determining leaf patterning and expansion, and influencing vascular development (Bashyal et al., 2024; Hirakawa, 2021; Somssich et al., 2016). The genes located at Chr2g0120661 and Chr2g0169701 encode putative CLV1 receptor and CLE plant peptide (**Supplementary Figure 4**). Typically, *CLV3* binds to *CLV1* to regulate a signaling cascade that normally enhances WUSCHEL-related homeobox (*WUS1* or *WOX*) gene expression (**Supplementary Figure 5**). CLE25 is a CLV3-like peptide whose direct target in roses might be CLV1 or another receptor like protein and was downregulated in rosetted tissues (**Figure 6D**). The striking opposite expression in symptomatic and asymptomatic tissue of *CVL1*, *WUS*, and *WOX1* underscores the critical role of these genes in the manifestation of rosetting disease (**Figure 6D**).

## Discussion

This study examined rosetting disease in cultivated roses grown in field plots. Infection occurred through natural transmission of RRV by its vector natural mite vector, *Phyllocoptes fructiphilus*. One of the most significant questions that has gone unanswered until now is whether the entire plant is uniformly infected with RRV, or whether infection only occurs in the rosetted tissues. Since RRV has seven genome segments, we employed novel qRT-PCR primers detecting viral RNA3, RNA5, RNA6, and RNA7 to learn that the viral RNA levels were significantly different in rosetted and asymptomatic tissues. Furthermore, the genome segments, which are typically packaged into virions, were not in equal amounts, which likely has strong implications for the development of disease. While unequal accumulation of viral RNA segments is not unusual, this is also reminiscent of a report by Sicard et al. (2019) revealed that faba bean necrotic stunt virus (FBNSV), which has eight single stranded DNA genome segments, were independently distributed in neighboring cells although the entire genetic material is encapsidated into a single particle. Further research is needed to examine genome packaging requirements as well as the distribution of viral genome segments in different cell types to better understand their implications for viral replication and disease development.

Another surprise was evidence that the homeostatic levels of CFA and GA1 were specifically altered in virus infected rosetted and non-rosetted leaves, while the levels of ABA and IAA in all tissues were comparable to healthy plants. GA1 levels in asymptomatic tissues were 3-fold or greater in asymptomatic tissues than rosetted or healthy tissues. CFA levels were suppressed in asymptomatic tissues compared to healthy, while elevated in symptomatic tissues compared to healthy. The importance of GAs as master regulator of plant growth promoting hormones was supported by the combined evidence that GA1 levels are higher in asymptomatic tissues and the preferential induction of GA metabolic factors in asymptomatic tissues. Promoter analysis of a subset of distinctly altered genes controlling plant hormone metabolism, revealed the abundance of GA responsive elements relative to *cis* elements responsive to other plant hormones. Regarding GA biosynthesis, we identified two genes encoding putative *ent*-kaurene synthases (Chr5g0023431 and Chr5g0023441) which convert *ent*-copalyl diphosphate into *ent*-kaurene (Wang et al., 2015) that were induced in asymptomatic tissues where GA1 levels were higher. Another gene encoding *ent*-kaurenoic acid oxidase (encoded by gene at Chr5g0014211) converts *ent*-kaurenoic acid to GA12, a precursor for GA1 and GA3, was downregulated in rosetted and asymptomatic tissues (Katyayini et al., 2020). GA12 is also a phloem mobile metabolite that can stimulate GA signaling cascade in distant tissues, controlling plant development (Hu et al., 2008; Regnault et al., 2015). Gibberellin 2-beta-dioxygenase (Chr5g0014211), which converts GAs to inactive forms, was downregulated in asymptomatic tissues leading to higher GA1 accumulation. Disruption of the GA biosynthetic pathway by specific viral proteins has been reported for barley yellow dwarf virus (BYDV), cauliflower mosaic virus (CaMV), RDV, and TMV-cg resulting in higher accumulation and exacerbated symptoms (Zhao and Li, 2021). For example, the P2 protein of RDV directly interferes with *ent*-kaurene oxidase leading to reduced GA1 levels causing abnormal leaves and dwarfing (Zhu et al., 2005). Barley yellow dwarf virus (BYDV) also interferes with GA accumulation, resulting in dwarfing symptoms (Russell, 1971). Regarding RRV, evidence that GA levels were elevated in rose tissues where RRV levels were lower suggests that the mode of GA action or its crosstalk mechanisms with other hormones correlate with virus levels and RRD symptoms.

RRV joins a growing list of positive and negative strand RNA viruses that alter the expression of certain genes controlling phytohormone biosynthesis, catabolism, or signaling. The results of this study suggest a model in which RRV directly or indirectly interferes with the promoter activities of genes involved in plant growth and hormone metabolism. With respect to auxin metabolism, we identified 16 genes that are specifically altered due to RRV infection with only seven that are precisely influenced by higher or lower levels of viral RNA accumulating in the tissues. These seven encode two crucial factors for auxin biosynthesis, named genes identified as Chr7g0192931 which encodes the putative ER-localized tryptophan aminotransferase-related protein 1 (*TAR1*) gene and Chr4g0422151 which encodes the putative *YUCCA10*. There were five genes linked to auxin catabolism transport, and transcriptional regulation: *IAGlu*, *PIN1*, *PILS3*, and two *SAUR* genes. Several studies over the past decade have shown that viral effectors can hijack transcriptional regulators in a manner that promotes their ability to spread throughout the plant. Examples include the NSs protein of TSWV, the Barley stripe mosaic virus (BSMV) γb, and rice stripe virus (RSV) NS3 proteins, bind to the TCP17 transcription factor (Zhao et al., 2024). The TCP class of transcription factors are widely essential for plant growth and development, regulating plant hormone metabolic gene expression, including those involved in auxins, ABA, GA, JA and SA pathways (Lopez et al., 2015). Moreover, the ability of TSWV, a close relative to RRV, to hijack TCP17 was shown to profoundly alter the expression of several *YUCCA* genes, thereby negatively impacting the endogenous auxin levels and signaling response pathways, resulting in plant dwarfing and distorted leaves (Zhao et al., 2024). An alternative model to direct viral protein binding of a transcription factor is represented by potyviruses and geminiviruses whose silencing suppressor proteins alter DNA methylation at the promoters of several *AtYUCCA* genes as well as genes involved in salicylic acid metabolism. The direct result of auxin overproduction is credited with severe symptoms including deformed inflorescences and severe leaf curling (Yang et al., 2020).

RRV infection causes massive changes in growth in mature tissues on one side of a plant, suggesting that the timing of new growth and development, plastochron, is uneven between regions of low and high virus titer. Age-related plant susceptibility to virus infection has been reported for decades, and only recently have scientists begun to understand that the abundance of certain age-related host factors can play a role in virus-host interactions, driving the spread of infection. For example, in mature tobacco plants, TMV stimulates the accumulation of AUX/IAA, which is a repressor of ARF transcription factors (Padmanabhan et al., 2008). The TMV 126/183 kDa protein hijacks AUX/IAA to enhance its spread in older tissues and causes physiological disorders in mature tobacco plants. Perhaps it is the movement of AUX/IAA by the 126/183 kDA protein in the phloem that leads to changes in phloem development, which promotes virus phloem loading and transport (Collum et al., 2016; Kappagantu et al., 2020). In roses that produce new shoots and branches from lateral buds, the phloem forms during cambial development and over time connects to the major stem of the plant. Typically, the vasculature of different canes within the same shrub are not connected through the stem, which allows for pruning to be performed without killing the entire plant (Pashina, 2021; Zieliński et al., 2010). It is worth speculating that RRV necessarily induces changes in the expression of factors such as TAR1, YUCCA, IAGlu, PIN1, PILS3, AUX/IAA, and SAUR proteins in order to enhance its own phloem transport and spread throughout mature tissues in the host plant.

These auxin-related factors are also controlled by crosstalk with CKs, ABA, SLs, and GAs through fine-tuned interactions (Béziat et al., 2017; Feraru et al., 2019; Martinez et al., 2016). ABA and CKs regulate degradation, phosphorylation, and distribution of PIN1 and PILS to direct auxin flow toward the growing axis (Feraru et al., 2022; Marhavý et al., 2014; Šimášková et al., 2015). SLs can interfere with PILS and PIN polarity and auxin trafficking in a manner that influences vascular tissue formation and shoot branching (Bennett et al., 2016; Bennett and Leyser, 2014). GAs can promote PIN1 degradation, influence its trafficking and influence auxin transport (Willige et al., 2011). In **Figures 5** and **6**, we identified a limited number of candidate genes that are likely direct targets for viral interference either by direct protein-protein interactions or suppression of gene silencing. In **Figure 6D**, we present a model comprising 13 genes that are critical regulatory factors in new growth occurring in mature plants and whose changing expression patterns are widely known to alter leaf development, branching and flowering. Thus, we propose that RRV proteins directly interfere with key regulatory proteins or silencing suppressors to alter gene expression patterns in a manner that enables the virus to capitalize on essential features to enhance its ability to spread and in mature plants, these cause abnormal growth.

We report potential disruption of normal ABA biosynthesis and ABA signaling in rosetted tissues as indicated by changes in the expression of the genes involved in these processes. ABA normally contributes to antiviral resistance through its ability to modulate callose deposition at plasmodesmata and increase expression of RNA-silencing genes such as *AGO1, AGO2*, and *AGO3* (Alazem et al., 2020, 2019, 2017; Alazem and Lin, 2020). Combining aphid herbivory with TuMV infection led to even greater changes in ABA-responsive genes and influenced alternative splicing of mRNAs (Herath et al., 2025). In the case of RRV infected roses, ABA signaling might be stimulated by the combination of insect herbivory and virus infection, thereby causing severe symptoms in only a portion of the plant where both were present. Alternative RRV proteins might directly interact with the two key ABA signaling molecules identified in **Figure 5**, hijacking them for pro-viral functions.

We identified a core set of genes that are differentially regulated in rosetted tissues, which likely control hyper-prickle development. Prickle development in many *Solanaceae* has been linked to the single dominant *LOG* gene, which encodes an ancient cytokinin activating enzyme (Satterlee et al., 2024). Prickle development and their lignification in *Rosa* sp*ecies* is a multigene trait controlled by CKs, GAs, and JA regulating the MBW transcriptional activator-inhibitor complex (Huang et al., 2023; Rawandoozi et al., 2024; Swarnkar et al., 2021; Zhou et al., 2021). Induction or suppression of *GL1* was expected to result in similar changes in *GL2* or *MYB123*, however the opposite occurred in rosetted tissues. Given that the samples were taken from leaves rather than stems, it is possible that had we developed a transcriptome dataset from rosetted stems, the outcomes among DEGs would be different. However, it is also possible that the data obtained in this study points to another example where plant viral proteins might directly interfere with normal signaling processes and independently direct changes in gene expression.

The CUC regulatory network is induced in symptomatic and asymptomatic tissues. While component factors influencing *CUC* gene expression appear to be dysregulated by virus infection, the current data do not readily point to this network as the underlying cause of rosetting disease. The co-regulation of *LOB*, *KNOX2*, and *CUC*s in this study is expected during normal leaf development (Maugarny et al., 2016; Spinelli et al., 2011). RAX1, 2, and RAX3 are redundant inducers of *CUC1/2/3* and their downregulation suggests that this route of *CUC* induction is not the primary driver for normal leaf development in asymptomatic tissues. If we consider a model in which *PIN1* localization and auxin transport are altered in rosetted tissues, it becomes difficult to imagine how the *CUC* regulatory network underlies the abnormal leaf morphology. Perhaps changes in cellular behavior during development in rosetted tissues involve more complex interactions involving miRNAs or brassinosteroids (BRs), which are also known to influence organ boundary formation in leaves and meristems (Maugarny et al., 2016; Spinelli et al., 2011; Sun et al., 2020). BRs signaling is vital for plant meristem and leaf development and can have pro-viral or antiviral activities. Exogenous application of BR enhances resistance to CMV, TMV and RSV in their host plants but reduces RBSDV resistance in rice (Hu et al., 2020; Zolkiewicz and Gruszka, 2022).

*WUS* and *CLV1* are crucial to leaf development as well as meristem development (Jha et al., 2020; Somssich et al., 2016). As seen in **Figure 6**, downregulation of *CLV3* has the opposite effect on WUS expression as they form a feedback loop. However, overexpression of *WUS* and *CLV1* can result in abnormal cell dedifferentiation, leaf development, and adventitious shoots, among other morphological changes. Based on current knowledge of *WUS*, *CLV1*, the higher expression of these genes in symptomatic tissue could be key drivers of the rosetting phenotype. *CLAVATA* signaling is linked to environmental interactions, but there is a lack of data showing CLAVATA signaling is linked to pathogen defenses, although there is insufficient evidence linking it to virus infection. On the other hand, *WUS* is known to act in anti-viral defenses inhibiting viral protein synthesis by repressing plant S-adenosyl-I-methioinine-dependent methyltransferases in order to exclude viruses from meristematic cells (Wu et al., 2020).

In conclusion, this system-level approach revealed an intricate coordination of plant hormone signaling networks underlying molecular and genetic mechanisms that control plant form and function, giving rise to rosetting disease. High success in mapping transcripts from infected garden roses to the *Rosa chinensis* genome was reflective of reported success in QTL mapping studies and ongoing marker-assisted selection efforts to identify the genetic basis of RRD resistance in hybrid roses using the same reference genome (Hochhaus et al., 2023; Lau et al., 2022; Rawandoozi et al., 2024a; Young et al., 2022). A large number of DEGs were divided into asymptomatic and symptomatic datasets for comparison, revealing that only 39 of these genes were oppositely expressed (up versus down) in these datasets. Combining information provided by the gene descriptions in the GDR database and GO analysis with family level phylogenetic analyses presented in the supplementary files, enabled us to elaborate the gene network models presented alongside heat maps in order to link gene expression changes to the RRD phenotype (Rawandoozi et al., 2024; Windham et al., 2023). The biological networks identified in this study by comparing rosetted, asymptomatic and healthy tissues revealed important signaling hubs that differentiate disease outcomes. Differential gene expression analysis also linked hormones such as SL and PAA that were not directly measured in our primary assessment to rosette disease although the transcriptomic dataset suggests that their levels are also altered. A much larger study will be needed to be performed with controlled inoculations and a time course of tissues sampling in greenhouse grown plants to better characterize the wider set of hormones and their fluctuating levels in various tissues that were not sampled (stems, petioles, roots, buds, flowers) in order to better understand how viral RNA levels broadly and precisely influence hormonal processes. Future investigations will link plant hormones such as salicylic and jasmonic acid, viral proteins, and key molecular drivers of host responses that serve to limit infection and disease.

## Data Availability

The datasets generated during this study are available at the NCBI SRA (PRJNA1285908). All other data are available upon request.

## Glossary

ABA: Abscisic acid
Asympt: Asymptomatic
AUX/IAA: Auxin/Indole-3-Acetic Acid
CCDs: Carotenoid cleavage dioxygenases
CK: Cytokinin
CLV: CLAVATA
CMV: Cucumber mosaic virus
COMT: Caffeic acid 3-O-methyltransferases
CUC: Cup shaped cotyledon
DEGs: Differentially expressed genes
DMAPP: Dimethylallyl pyrophosphate
ER: Endoplasmic reticulum
FDR: False discovery rate
GA: Gibberellin
GAPC: Glyceraldehyde 3-phosphate
GDR: Genome database for Rosaceae
GFP: Green fluorescent protein
GGPP: Geranylgeranyl diphosphate
GH3: Gretchen Hagen 3
GL1: GLABRA1
GL2: GLABRA2
GL3: GLABRA3
Glu-trans: Glutamic acid transferase
GO: Gene ontology
GRV: Groundnut rosette virus
IAA: Indole 3 acetic acid
ICTV: International Committee on Taxonomy of Viruses
IPP: Isopentenyl pyrophosphate
LOB: Lateral organ boundary
LOF1: Lateral organ fusion 1
MEP: Methyl erythritol phosphate
NCED: 9-cis-epoxycarotenoid dioxygenases
ORFs: Open reading frames
PAA: phenylacetic acid
PILs: PIN-LIKES
PIN1: PIN-FORMED 1
PYR: Pyrabactin Resistance
RAX3: REGULATOR OF AXILLARY MERISTEMS3
RNA-seq: RNA sequencing
RRD: Rose rosette disease
RRV: Rose rosette virus
SAUR: small auxin up-regulated RNA
SAM: Shoot apical meristem
SHI: SHORT INTERNODES
SLs: Strigolactones
STM: SHOOTMERISTEMLESS
Sympt: Symptomatic
VLPs: Virus like particles
WUS: WUSCHEL
TTG1: TRANSPARENT TESTA GLABRA1
TTG2: TRANSPARENT TESTA GLABRA2
TT2: TRANSPARENT TESTA 2
TuRV: Turnip rosette virus
