# Supplementary material for: Distinct gene reprogramming in rosetted and symptomless shoots from the same mature rose plants infected with rose rosette virus
Source: Front Plant Sci. 2025 Aug 28;16:1635660. doi: 10.3389/fpls.2025.1635660 (PMC12423424; doi:10.3389/fpls.2025.1635660)
Supplement: Supplementary file 1 [file DataSheet1.pdf]

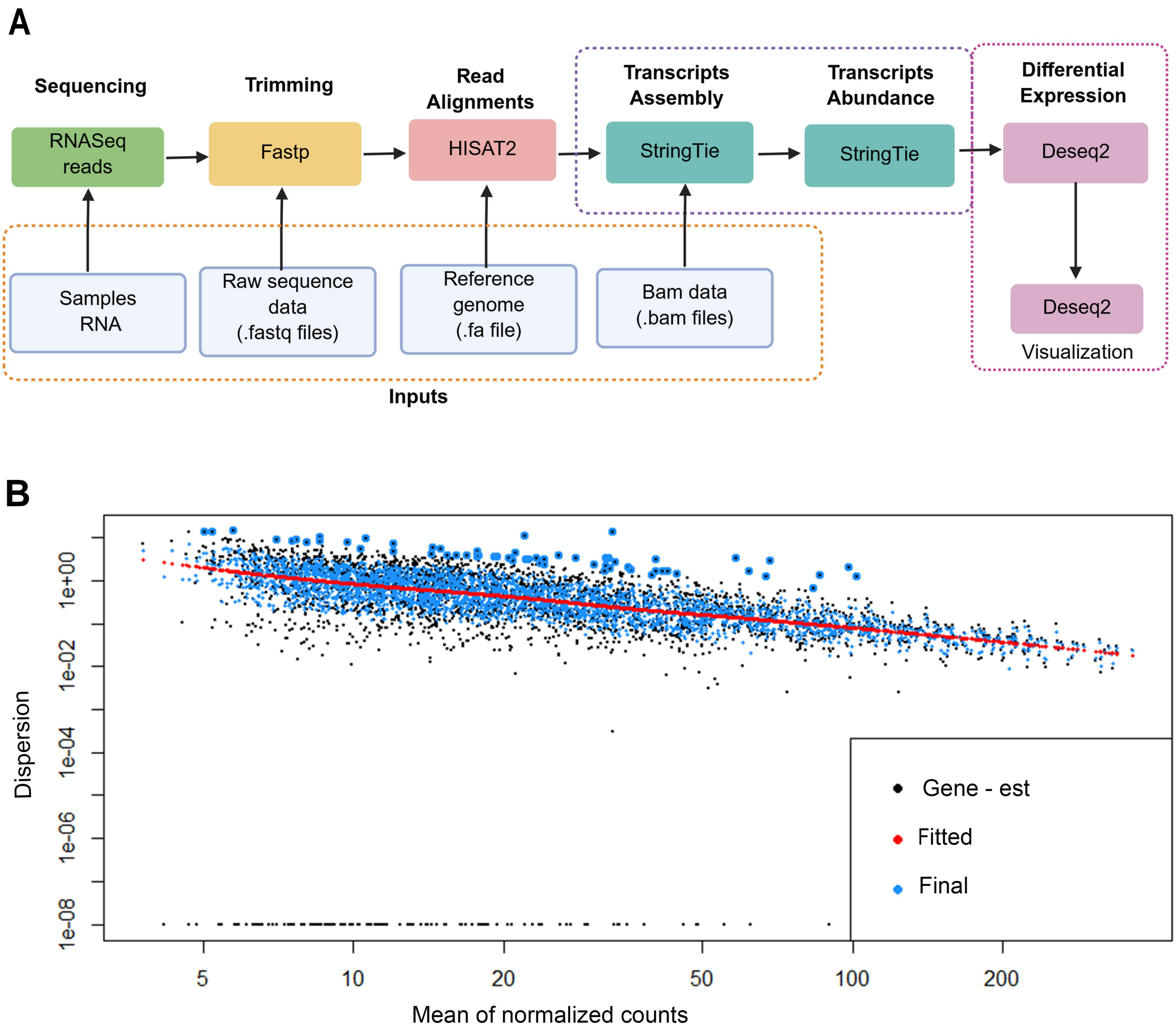


Figure S1. (A) RNA-seq data analysis pipeline and (B) dispersion plot representing the spread of data within the datasets. The estimated gene clustering and dispersal are presented as black and blue dots along the red fitted trend line. The Y - axis represents genes dispersion estimate. X – axis represents gene expression level after normalization.

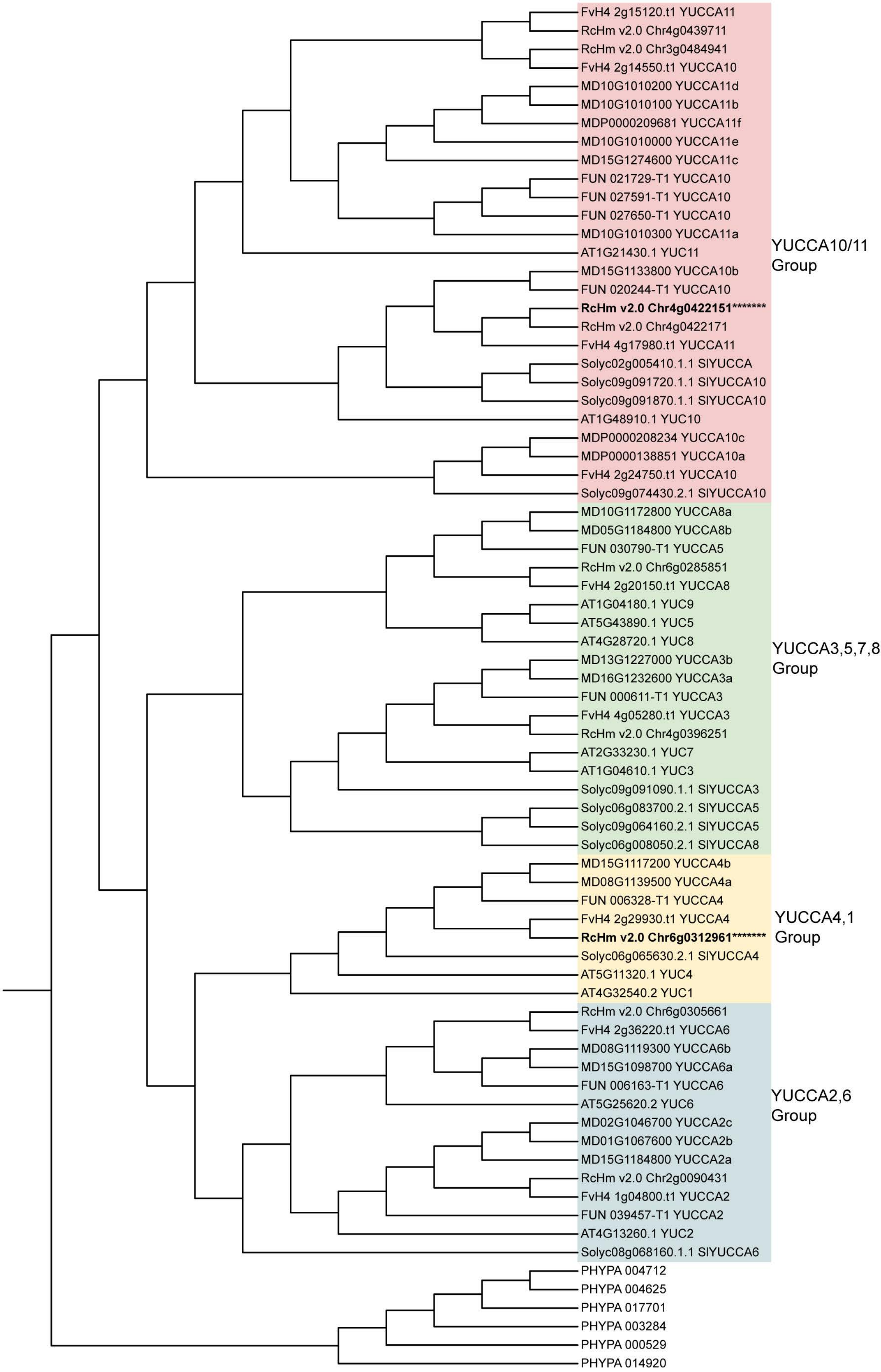


Figure S2. Phylogeny of RcYUCCA auxin biosynthetic proteins. YUCCA family expansion across plant species occurred by gene duplication and functional diversification over evolution influenced by selection imposed by promoters restricting gene expression to certain cell types (Expósito-Rodríguez et al., 2011; Hentrich et al., 2013; H. Liu et al., 2014; Meng et al., 2023; Munguía-Rodríguez et al., 2020). The selection pressures imposed on coding region validates using phylogeny to infer shared functions.Thus, gene variants in a phylogeny may be closely related, implying functional redundancy, and at the same time contain promoter elements that may compound environmental stress response, hormone responsiveness with cell-type restrictions. ML phylogenetic trees constructed using YUCCA genes belonging to the following plant species: *Arabidopsis thaliana, Fragaria vesca* (strawberry), *Malus domestica* (apple), *Prunus avium* (almond), *Physocmitrium patens*, *Solanum lycopersicum* (tomato) (Expósito-Rodríguez et al., 2011; Hentrich et al., 2013; H. Liu et al., 2014; Song et al., 2020). Protein names and encoding gene IDs are provided in the tree. The functions of *A. thaliana, S. lycopersicum,* and *P. patens* YUCCAs have been studied in numerous reports making them useful for phylogenetic analysis to infer shared functions (Cao et al., 2019; Expósito-Rodríguez et al., 2011; Meng et al., 2023; Thelander et al., 2018). The phylogeny tree shows one or more variants of each YUCCA factor often cluster with single *A. thaliana* factors. Color blocks highlight four major clades to make this presentation easier for the reader to examine. There are two RcYUCCA factors clustering with AtYUC11, two RcYUCCA clustering with AtYUC10, one RcYUCCA clustering with AtYUC5/8/9, one RcYUCCA clustering with AtYUC3/7, one RcYUCCA clustering with AtYUC1/4, and two RcYUCCA clustering with AtYUC2/6. The RcYUCCA4 and RcYUCCA10 are identified among the DEGs in this study are identified in bold with "******" and were named according to their predicted relatedness to AtYUC and SlYUC factors.

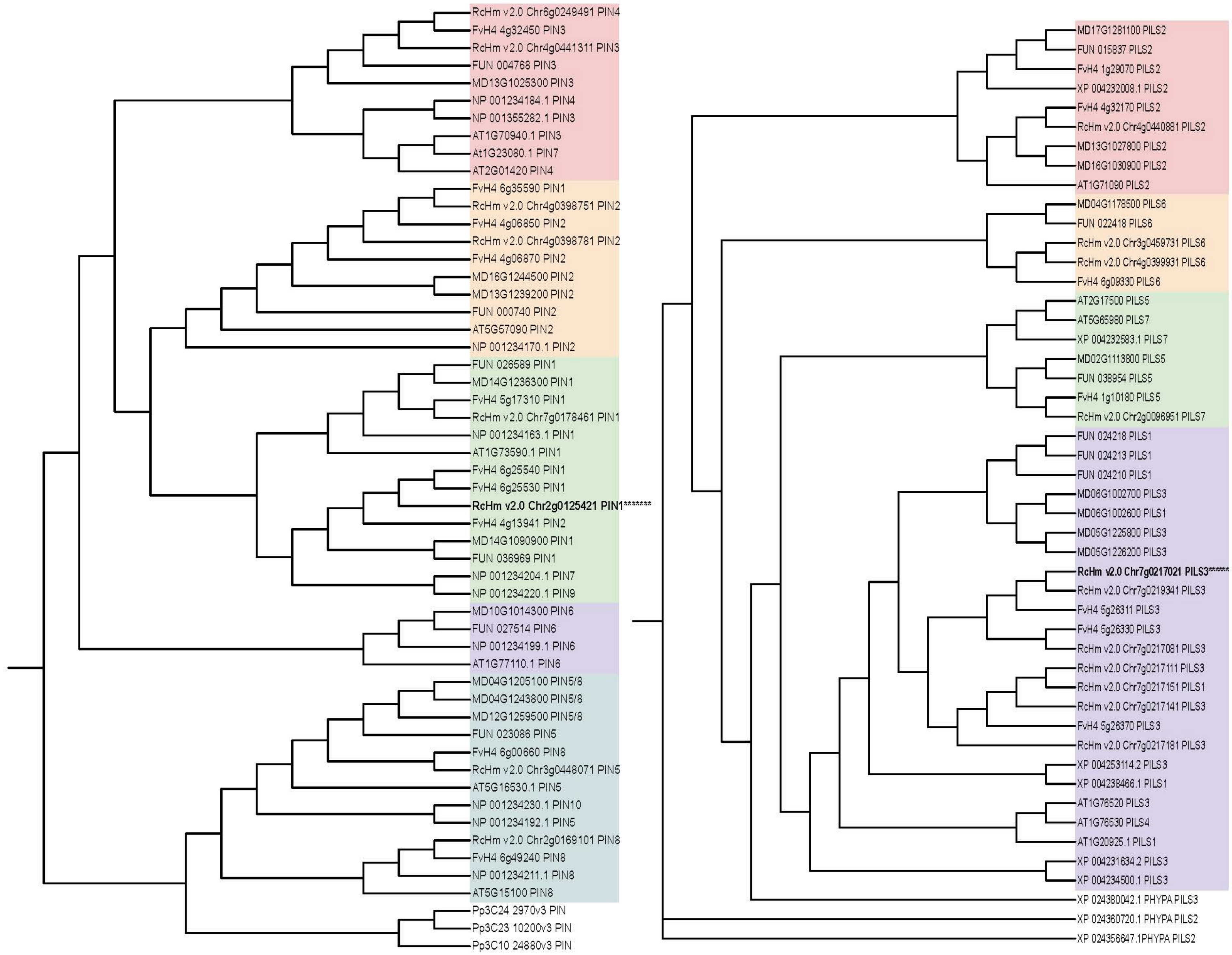


Figure S3. Phylogeny of PIN-FORMED (PIN) and PIN-Formed Likes (PILS) proteins was performed as per the justification detailed in Figure S2 to understand the family relatedness and infer shared functions. ML phylogenetic trees constructed using PIN gene sequence and PILs from *R. chinensis* (rose), *M. domestica* (apple), *F. vesca* (strawberry), *P. avium* (almond), *S. lycopersicum* (tomato), *A. thaliana*, and *P. patens* (moss). Protein names and encoding gene IDs are provided in the tree. The functions of members of the *A. thaliana, S. lycopersicum, and P. patens* PIN and PILS family have been studied making them useful for phylogenetic analysis to infer shared functions (Lüth et al., 2023; Sauer & Kleine-Vehn, 2019; Šimášková et al., 2015; Sun et al., 2020; Willige et al., 2011; Zhou & Luo, 2018). The major clades for PIN (left) and PILs (right) phylogenetic trees are color coded. *R. chinensis* has six PIN genes and two are found in a major clade with PIN1 factors of other species (Martinez et al., 2016; Pattison & Catalá, 2012). *R. chinensis* also has 11 PILS factors distributed across four major clades highlighted by color blocks. A particular clade highlighted in purple derives from StPILS1/3 factors and AtPILS1/3/4 factors with seven RcPILS factors. This clade has several *Rosaceae* species for which the gene homologues have been named PILS1 or PILS3 in the GDR database. This phylogeny suggests further evolutionary and functional studies of the PILS gene family is needed to improve nomenclature and clarify gene functions. The seven RcPILS1/3 factors in this clade derive from chromosome 7, and probably have functional redundancy as well as unique features, and that their promoter elements have divergent roles in environmental stress response, hormone responsiveness, and cell-type restrictions (B. Liu et al., 2014; Willige et al., 2011). This is a topic for another investigation. The RcPIN1 and RcPILS3 identified among the differentially expressed genes are identified in bold with "*******", respectively.

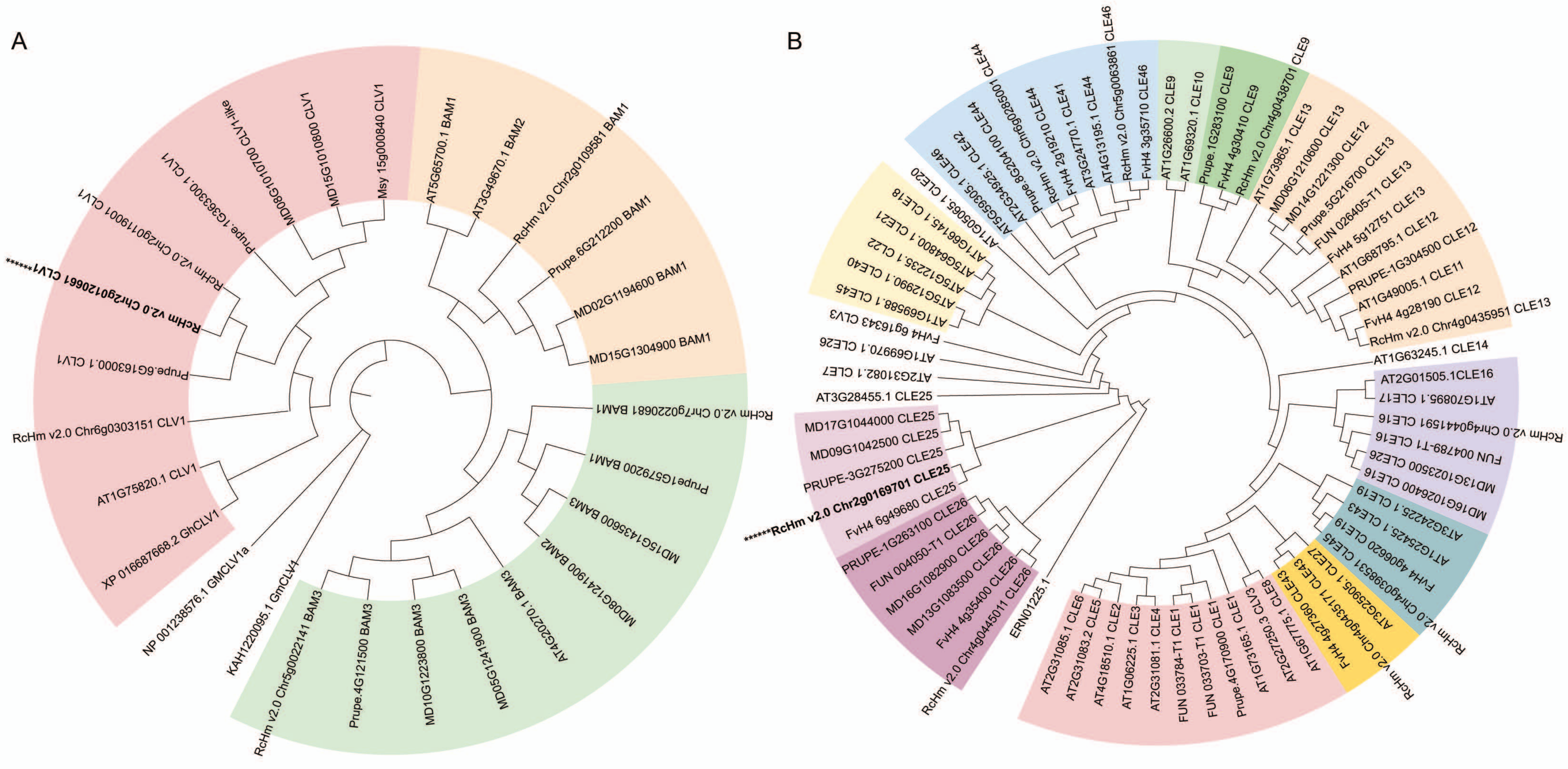


Figure S4. (A) Phylogeny of CLAVATA1 (CLV1) and BARELY ANY MERISTEMS (BAM1,2,3) comprise the CLV-family (CLVf) of receptor like kinases (RLKs) that perceive CLAVATA3/EMBRYO SURROUNDING REGION (ESR)-RELATED (CLE) family peptide signals (B) (Bashyal et al., 2024; Fiers et al., 2006; Hirakawa, 2021). Members of these CLV/BAM gene families (A) and CLE (B) gene families are recently reported and more are likely to be discovered. Protein names and encoding gene IDs are provided in the trees. (A) The CLV1 circular phylogenetic trees revealed three clades with CLV1 distinct from BAM1,2 and 3 genes.
The gene encoding RcCLV1 is identified in bold with "******". (B) In *A. thaliana* there are at least 45 CLE family members in addition to CLV3 (Fiers et al., 2006; Hirakawa, 2021; Somssich et al., 2016). Across Rosaceae the nomenclature for CLE family members is not so precise and may be linked to suggested relatedness to an *A. thaliana* gene based on limited phylogenetic information. We identified a gene encoding a putative CLE25 which resides in a clade with other *Rosaceae* and *Arabidopsis* CLE25 as their nearest relatives. The gene encoding RcCLE25 is identified in bold with "******".

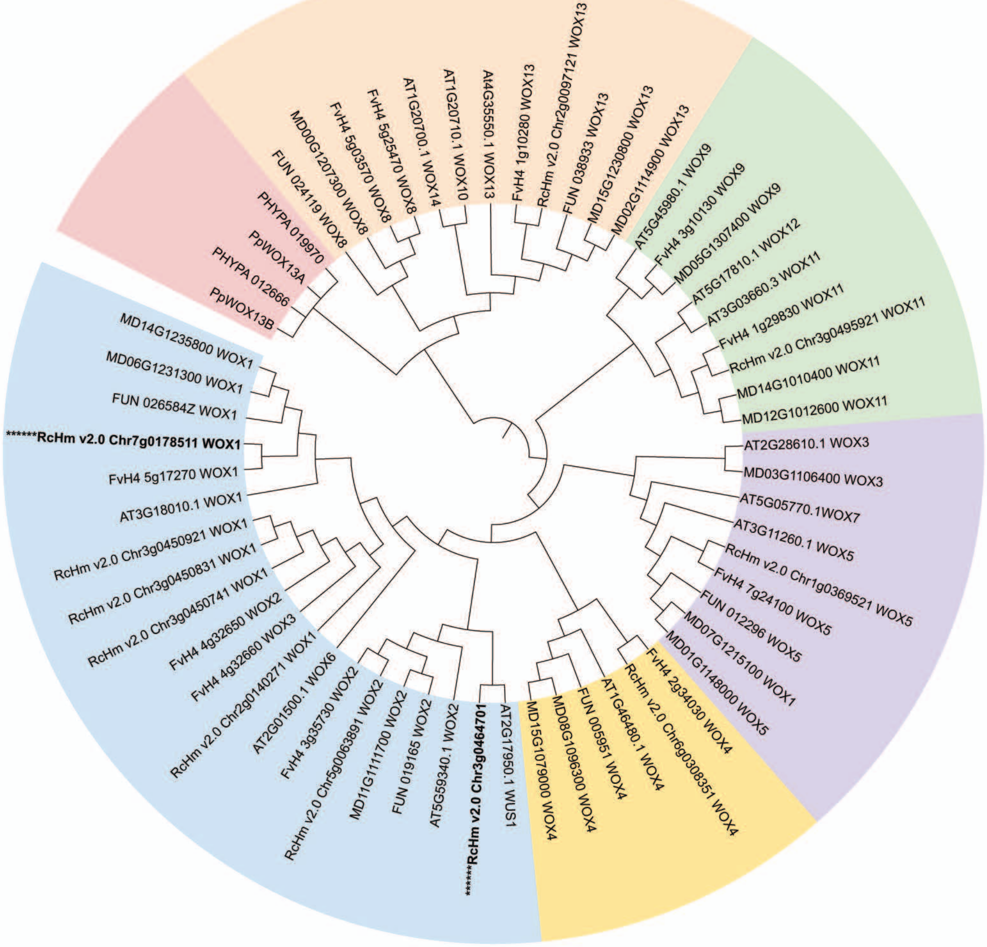


Figure S5. ML phylogenetic trees constructed using WUSCHEL (WUS) and WUSHEL -homeobox-like (WOX) genes encode transcripton factors that positively regulate expression of CLV3 and is required for the regulation of embryogenec and meristematic stem cells (Jha et al., 2020; Somssich et al., 2016). WUS and WOX are central to plant growth and development. WUS is differentially expressed in the shoot apical meristem, controlling shoot, floral, and reproductive organ development. WOX genes control embryo development, vascular development, lateral organ development including leaves and branches (Costanzo et al., 2014; Deveaux et al., 2008; Rasheed et al., 2024). The circular tree highlights six clades containing homologs from multiple species suggesting evolutionary relationships. The blue clade has seven *R. chinensis* genes labeled as WOX1, WOX2, or not given a label according to the GDR database. The phylogeny showed a single RcWUS1 closely related to AtWUS1 and another RcWOX1 related to AtWOX1 which are represented among the DEGs. These are identified in bold with “******”.

**Supplementary References**

Bashyal, S., Gautam, C. K., & Müller, L. M. (2024). CLAVATA signaling in plant–environment interactions. Plant Physiol. 194, 1336–1357. https://doi.org/10.1093/plphys/kiad591.

Cao, X., Yang, H., Shang, C., Ma, S., Liu, L., & Cheng, J. (2019). The roles of auxin biosynthesis YUCCA gene family in plants. Internat. J. Molec. Sciences.20,1-18. https://doi.org/10.3390/ijms20246343.

Costanzo, E., Trehin, C., & Vandenbussche, M. (2014). The role of WOX genes in flower development. Annals of Botany. 114, 1545–1553. https://doi.org/10.1093/aob/mcu123.

Deveaux, Y., Toffano-Nioche, C., Claisse, G., Thareau, V., Morin, H., Laufs, P., Moreau, H., Kreis, M., & Lecharny, A. (2008). Genes of the most conserved WOX clade in plants affect root and flower development in Arabidopsis. BMC Evolutionary Biology, 8(1): 1-18. https://doi.org/10.1186/1471-2148-8-291.

Expósito-Rodríguez, M., Borges, A. A., Borges-Pérez, A., & Pérez, J. A. (2011). Gene structure and spatiotemporal expression profile of tomato genes encoding YUCCA-like flavin monooxygenases: The ToFZY gene family. Plant Physiol. and Biochem., 49(7), 782–791. https://doi.org/10.1016/j.plaphy.2011.02.022.

Fiers, M., Golemiec, E., Van Der Schors, R., Van Der Geest, L., Li, K. W., Stiekema, W. J., & Liu, C. M. (2006). The CLAVATA3/ESR motif of CLAVATA3 is functionally independent from the nonconserved flanking sequences. Plant Physiology, 141(4), 1284–1292. https://doi.org/10.1104/pp.106.080671.

Hentrich, M., Sánchez-Parra, B., Marta-Marina, P. A., Loba, V. C., Carrillo, L., Vicente-Carbajosa, J., Medina, J., & Pollmann, S. (2013). YUCCA8 and YUCCA9 overexpression reveals a link between auxin signaling and lignification through the induction of ethylene biosynthesis. Plant Signaling and Behavior, 8(11): 1-4. https://doi.org/10.4161/psb.26363.

Hirakawa, Y. (2021). CLAVATA3, a plant peptide controlling stem cell fate in the meristem. Peptides. 142: 1-7. https://doi.org/10.1016/j.peptides.2021.170579.

Jha, P., Ochatt, S. J., & Kumar, V. (2020). WUSCHEL: a master regulator in plant growth signaling. Plant Cell Reports. 39:431–444. https://doi.org/10.1007/s00299-020-02511-5.

Liu, B., Zhang, J., Wang, L., Li, J., Zheng, H., Chen, J., & Lu, M. (2014). A survey of Populus PIN-FORMED family genes reveals their diversified expression patterns. J. Experi. Botany. 65: 2437–2448.https://doi.org/10.1093/jxb/eru129.

Liu, H., Xie, W. F., Zhang, L., Valpuesta, V., Ye, Z. W., Gao, Q. H., & Duan, K. (2014). Auxin Biosynthesis by the YUCCA6 flavin monooxygenase gene in woodland strawberry. J. Integ. Plant Biology. 56(4): 350–363. https://doi.org/10.1111/jipb.12150.

Lüth, V. M., Rempfer, C., van Gessel, N., Herzog, O., Hanser, M., Braun, M., Decker, E. L., & Reski, R. (2023). A Physcomitrella PIN protein acts in spermatogenesis and sporophyte retention. New Phytologist, 237(6), 2118–2135. https://doi.org/10.1111/nph.18691.

Martinez, C. C., Koenig, D., Chitwood, D. H., & Sinha, N. R. (2016). A sister of PIN1 gene in tomato (Solanum lycopersicum) defines leaf and flower organ initiation patterns by maintaining epidermal auxin flux. Developmental Biology, 419(1), 85–98. https://doi.org/10.1016/j.ydbio.2016.08.011.

Meng, S., Xiang, H., Yang, X., Ye, Y., Ma, Y., Han, L., Xu, T., Liu, Y., Wang, F., Qi, M., & Li, T. (2023). Analysis of YUC and TAA/TAR gene families in tomato. Horticulturae, 9(6): 1-13. https://doi.org/10.3390/horticulturae9060665.

Munguía-Rodríguez, A. G., López-Bucio, J. S., Ruiz-Herrera, L. F., Ortiz-Castro, R., Guevara-García, Á. A., Marsch-Martínez, N., Carreón-Abud, Y., López-Bucio, J., & Martínez-Trujillo, M. (2020). YUCCA4 overexpression modulates auxin biosynthesis and transport and influences plant growth and development via crosstalk with abscisic acid in Arabidopsis thaliana. Genetics and Mol. Biology, 43(1): 1-14. https://doi.org/10.1590/1678-4685-GMB-2019-0221.

Pattison, R. J., & Catalá, C. (2012). Evaluating auxin distribution in tomato (Solanum lycopersicum) through an analysis of the PIN and AUX/LAX gene families. Plant J. 70(4), 585–598. https://doi.org/10.1111/j.1365-313X.2011.04895.x.

Rasheed, H., Shi, L., Winarsih, C., Jakada, B. H., Chai, R., & Huang, H. (2024). Plant growth regulators: An overview of WOX gene family. Plants 13(21): 1-16. https://doi.org/10.3390/plants13213108.

Sauer, M., & Kleine-Vehn, J. (2019). PIN-FORMED and PIN-LIKES auxin transport facilitators. Development (Cambridge), 146(15): 1-5. https://doi.org/10.1242/dev.168088.

Šimášková, M., O'Brien, J. A., Khan, M., Van Noorden, G., Ötvös, K., Vieten, A., De Clercq, I., Van Haperen, J. M. A., Cuesta, C., Hoyerová, K., Vanneste, S., Marhavý, P., Wabnik, K., Van Breusegem, F., Nowack, M., Murphy, A., Friml, J., Weijers, D., Beeckman, T., & Benková, E. (2015). Cytokinin response factors regulate PIN-FORMED auxin transporters. Nature Communications, 6: 1-11. https://doi.org/10.1038/ncomms9717.

Somssich, M., Je, B. II, Simon, R., & Jackson, D. (2016). CLAVATA-WUSCHEL signaling in the shoot meristem. Development 143: 3238–3248. https://doi.org/10.1242/dev.133645.

Song, C., Zhang, D., Zheng, L., Shen, Y., Zuo, X., Mao, J., Meng, Y., Wu, H., Zhang, Y., Liu, X., Qian, M., Zhang, J., Li, G., Zhao, C., Xing, L., Ma, J., Han, M., & An, N. (2020). Genome-wide identification and expression profiling of the YUCCA gene family in Malus domestica. Scientific Reports, 10(1): 1-12. https://doi.org/10.1038/s41598-020-66483-y.

Sun, L., Feraru, E., Feraru, M. I., Waidmann, S., Wang, W., Passaia, G., Wang, Z. Y., Wabnik, K., & Kleine-Vehn, J. (2020). PIN-LIKES coordinate brassinosteroid signaling with nuclear

auxin input in Arabidopsis thaliana. Current Biology, 30(9)1579-1588.e6. https://doi.org/10.1016/j.cub.2020.02.002.

Thelander, M., Landberg, K., & Sundberg, E. (2018). Auxin-mediated developmental control in the moss *Physcomitrella patens*. J. Experi. Botany 69 (2): 277–290. https://doi.org/10.1093/jxb/erx255

Willige, B. C., Isono, E., Richter, R., Zourelidou, M., & Schwechheimer, C. (2011). Gibberellin regulates PIN-FORMED abundance and is required for auxin transport-dependent growth and development in Arabidopsis thaliana. Plant Cell, 23(6): 2184–2195. https://doi.org/10.1105/tpc.111.086355.

Zhou, J. J., & Luo, J. (2018). The PIN-FORMED auxin efflux carriers in plants. In International J. Molec. Sciences. 19(9): 1-21. https://doi.org/10.3390/ijms19092759.
